# Location selection for offshore wind power station using interval-valued intuitionistic fuzzy distance measure-RANCOM-WISP method

**DOI:** 10.1038/s41598-024-54929-6

**Published:** 2024-02-27

**Authors:** Pratibha Rani, Arunodaya Raj Mishra, Fausto Cavallaro, Adel Fahad Alrasheedi

**Affiliations:** 1 Department of Engineering Mathematics, Koneru Lakshmaiah Education Foundation, Guntur, Andhra Pradesh 522302 India; 2 Department of Mathematics, Government College Raigaon, Satna, Madhya Pradesh 485441 India; 3 Department of Economics, University of Molise, Via De Sanctis, 86100 Campobasso, Italy; 4 Statistics and Operations Research Department, College of Science, King Saud University, Riyadh, 11451 Saudi Arabia

**Keywords:** Offshore wind power station, Location selection, Interval-valued intuitionistic fuzzy set, Distance measure, RANCOM, WISP, Wind energy, Computational science, Socioeconomic scenarios, Sustainability

## Abstract

The development opportunities and high-performance capacity of offshore wind energy project depends on the selection of the suitable offshore wind power station (OWPS) location. The present study aims to introduce a decision-making model for assessing the locations for OWPS from multiple criteria and uncertainty perspectives. In this regard, the concept of interval-valued intuitionistic fuzzy set (IVIFS) is utilized to express uncertain information. To quantify the degree of difference between IVIFSs, an improved distance measure is proposed and further utilized for deriving the objective weights of criteria. Numerical examples are discussed to illustrate the usefulness of introduced IVIF-distance measure. The RANking COMparison (RANCOM) based on interval-valued intuitionistic fuzzy information is presented to determine the subjective weights of criteria. With the combination of objective and subjective weights of criteria, an integrated weighting tool is presented to find the numeric weights of criteria under IVIFS environment. Further, a hybrid interval-valued intuitionistic fuzzy Weighted integrated Sum Product (WISP) approach is developed to prioritize the OWPS locations from multiple criteria and uncertainty perspectives. This approach combines the benefits of two normalization tools and four utility measures, which approves the effect of beneficial and non-beneficial criteria by means of weighted sum and weighted product measures. Further, the developed approach is applied to the OWPS location selection problem of Gujarat, India. Sensitivity and comparative analyses are presented to confirm the robustness and stability of the present WISP approach. This study provides an innovative decision analysis framework, which makes a significant contribution to the OWPS locations assessment problem under uncertain environment.

## Introduction

Due to population growth and economic growth, global energy demand has been growing exponentially. Up to now, conventional sources of energy have been used and contributed to one-third of greenhouse gas emissions. The need for clean energy and its related services is increasing to fulfill the sustainable development goals. Renewable energy resources and technologies have potential to offer solutions for the long-term energy problems. Many countries have started to install facilities that use renewable energy resources^1^. Wind power is one of the most efficient, sustainable and long-standing renewable energy resources, which has the potential to tackle several current socio-economic and technological challenges. In the recent past, new policies combined with reduced costs and improved technologies have emerged as strong agent in the remarkable growth of wind energy market. The cost of wind energy production is expected to more affordable than fossil fuel-based energy^2,3^. Consequently, it is a clean renewable energy resource, which contributes significantly to the reduction of greenhouse gas emissions. As stated by the *International Renewable Energy Agency *(*IREA*), the world needs to install a minimum of 180 GW of new WP every single year to limit global warming and keep the global temperature increase below 2°C above pre-industrial levels^4^.

In the context of global issues facing against climate change, offshore wind power is key to the transition to a zero-carbon energy supply. The offshore wind power industry is rapidly increasing and will become one of the attentions of new energy development in the future^5^. Lower roughness coefficient and higher wind speeds on sea surface empower offshore wind energy to be taken into consideration for power generation. In the last decade, offshore wind installation has widely gained into momentum^6^. Offshore wind power has the high-power generation proficiency, less requirement of land resources and easy large-scale growth. The development and utilization of offshore wind energy requires a suitable location to establish the offshore wind power station (OWPS)^2,3,7,8^. A suitable location for OWPS can lessen the strain of construction and enable impending maintenance. It can expand the power generation capacity of OWPS together with the safe operations of wind turbines. Selecting the OWPS locations requires wide-ranging consideration of several dimensions such as economic, environmental, societal, technical, political, risk, organizational etc., therefore, this process can be considered as a multi-criteria decision-making (MCDM) problem^7,8^.

Because of the overwhelming vagueness and complexity of local and global environments together with the subjectivity of human’s mind, it is not always possible for the decision experts to express the evaluation value of an alternative in terms of crisp number. To conquer this issue, the theory of fuzzy set has been originated, by Zadeh^9^. As the fuzzy set only contains a membership degree, therefore, Atanassov^10^ extended the classical fuzzy set and investigated the notion of intuitionistic fuzzy set, which assigns a membership degree, a non-membership degree and an indeterminacy degree to each element with sum of the membership and non-membership degrees is bounded to 1. After the pioneering innovation by Atanassov^10^, various significant results have been achieved based on intuitionistic fuzzy set theory^11–13^. In the theory of intuitionistic fuzzy set, the representation of the membership and non-membership degrees are all exact numbers, which is tough for the decision experts to express their preference information in some real-life circumstances. To handle these situations, Atanassov and Gargov^14^ investigated the theory of interval-valued intuitionistic fuzzy set (IVIFS), which expresses the support, opposition and neutrality of decision-makers by the interval-valued membership, non-membership and indeterminacy degrees, respectively. Simultaneously, some relations and basic operations, score and accuracy functions have been presented for IVIFSs^15^. As an extended version of intuitionistic fuzzy set, the IVIFS theory provides a more useful and reasonable way to cope with imprecise and uncertain information. Due to its higher flexibility in dealing with fuzzy data, the IVIFS doctrine has been broadly explored from different perspectives. Few of them are listed as^2,3,16–18^. Now, it is necessary to mention some significant fact regarding neutrosophic sets in the sense that an IVIFS is a particular case of neutrosophic set and refined neutrosophic set^19^. According to the Smarandache^20^, “neutrosophic set is the wider notion of fuzzy sets, intuitionistic fuzzy sets, Pythagorean fuzzy sets, q-rung orthopair fuzzy sets, picture fuzzy sets, spherical fuzzy sets and n-Hyper spherical fuzzy set”. In view of the notion of neutrosophic sets, we can say that an IVIFS is a special case of neutrosophic set^21–24^ and refined neutrosophic set^20^. The basic difference between an IVIFS and neutrosophic set (refined neutrosophic set) is that the parameters (i.e., membership degrees) in an IVIFS are dependent whereas the components in a neutrosophic set (refined neutrosophic set) are independent of each other.

In the literature, various approaches have been developed to deal with the MCDM problems from interval-valued intuitionistic fuzzy information perspective^1–3,16–18,25,26^. In the recent past, Stanujkic et al.^27^ pioneered the concept of Weighted Integrated Sum Product (WISP) method combining the notions of weighted sum model and weighted product model. It employs four utility measures to compute the overall utility of options and rank the options in an easier way. The main difference between the WISP approach and other existing MCDM methods is in the way the data are normalized and how the final ranks of options are determined. Few authors have extended the classical WISP approach and applied to real-life decision-making problems^21,22,28–30^. So far, there is no research which develops the WISP approach under interval-valued intuitionistic fuzzy environment. As the use of only one normalization procedure could lead to imprecise decision, therefore, this study develops an integrated interval-valued intuitionistic fuzzy information-based WISP method to rank the OWPS locations, which includes two normalization procedures. Moreover, this study proposes an integrated weighting tool based on the distance measure and RANking COMparison (RANCOM) model under IVIFS environment. Here, the distance measure and RANCOM model are used to compute the objective and subjective weights of criteria, while the WISP method is applied to determine the ranking of OWPS locations. In the following, the key contributions of this paper are presented as follows:

- A new distance measure is proposed for IVIFSs, which avoids the limitations of existing interval-valued intuitionistic fuzzy distance measures^31–34^ by quantifying the difference between IVIFSs.
- To solve the MCDM problems with unknown criteria and decision experts’ weights, a novel extension of weighted integrated sum product method is proposed under interval-valued intuitionistic fuzzy environment.
- To derive the criteria weights, a hybrid weight-determination model is presented with the integration of introduced distance measure for objective weight and RANCOM model for subjective weight with interval-valued intuitionistic fuzzy information.
- The presented approach is applied on a case study of OWPS location selection problem, which proves its applicability and powerfulness.

The rest part of this study is organized as follows: "Literature review" section discusses the existing studies related to this work. "Distance measure for IVIFSs" section firstly confers the fundamental concepts and then introduces a new distance measure for IVIFSs. "A hybrid WISP method for interval-valued intuitionistic fuzzy MCDM problems" section develops a hybrid WISP methodology for assessing the multi-criteria OWPS locations under IVIFS context. "Result and discussion" section implements the proposed method on a case study of OWPS locations assessment. Furthermore, this section presents the sensitivity and comparative analyses. “Conclusions” section concludes the whole study and recommends for future researches.

## Literature review

This section presents the comprehensive literature related to the present work.

### MCDM methods for OWPS locations assessment

The MCDM techniques allow the best choice from a set of alternatives based on multiple and concurrent criteria. The application of MCDM models has gained more attention in the field of OWPS locations assessment. For instance, Fetanat and Khorasaninejad^35^ assessed the locations for offshore wind farm based on six criteria including depths, heights, proximity to facilities, economic, technical, environmental aspects. For this purpose, they presented a technique based on the combination of Analytic Network Process (ANP), Decision Making Trail and Evaluation Laboratory (DEMATEL) and Elimination et Choix Traduisant la Realité (ELECTRE) approaches with fuzzy information and applied to a real case study of offshore wind farm location selection in Iran. Wu et al.^36^ developed a novel intuitionistic fuzzy ELECTRE-III method for evaluating the offshore wind farm locations. In this model, the generalized intuitionistic fuzzy ordered weighted geometric interaction averaging operator has used to deal with the interaction between criteria. Wu et al.^37^ proposed a fuzzy MCDM method to evaluate the possible locations for offshore wind farm. They evaluated the locations by means of the feasibility of installation and maritime safety. Tercan et al.^38^ proposed a decision support system based on Geographic Information System (GIS) with its application in the assessment of bottom-fixed offshore wind farm locations in two different countries. They assessed the locations by considering the factors such as wind velocity, water depth, shorelines, fishing areas, shipping routes, environmental protection areas, transportation, and military zones. Abdel-Basset et al.^5^ noticed the shortcomings of existing methods for OWPS locations assessment. Further, they proposed a hybrid MCDM model with the integration of Analytical Hierarchy Process (AHP) and Preference Ranking Organization Method for Enrichment Evaluations (PROMETHEE)-II methods under the context of neutrosophic sets. Using the GIS, Genç et al.^39^ proposed a MCDM model for selecting the potential locations for OWPS in the coastal region of Turkey. They considered social, technical and environmental dimensions to evaluate the OWPS locations. Zhou et al.^40^ prioritized the OWPS locations using a hybrid MCDM model. For this purpose, they incorporated the Best Worst Method (BWM) and TOmada de Decisao Interativa Multicriterio (TODIM) approach with probabilistic linguistic term sets wherein the BWM has used to determine the criteria weights and integrated TODIM has utilized to rank the OWPS location alternatives. By means of 17 criteria, Caceoğlu et al.^41^ proposed a quantitative method for evaluating the OWPS locations in Northwest Turkey. In this regard, they integrated the GIS and AHP approaches to develop a hybrid decision-making model. With the use of two distance-based approaches, AHP and GIS, Sánchez-Lozano et al.^42^ evaluated the OWPS locations under fuzzy environment. To evaluate the OWPS locations, Wang et al.^4^ proposed a two-stage MCDM methodology combining the AHP and Weighted Aggregated Sum Product Assessment (WASPAS) methods with spherical fuzzy information. Karipoğlu et al.^8^ combined the GIS, AHP and Evaluation based on Distance Average Solution (EDAS) methods with fuzzy sets and applied to evaluate the hybrid offshore wind and solar power plant. Abdel-Basset et al.^7^ extended the EDAS method from neutrosophic information perspective to assess the OWPS locations. In addition, they used AHP model to compute the weights of considered factors in assessing the OWPS locations under neutrosophic environment. With the use of Pythagorean hesitant fuzzy information, Zhou et al.^2,3^ developed a model and presented its application in OWPS locations evaluation. For this purpose, they combined a subjective weighting model, named as Step wise weight assessment ratio analysis (SWARA) and a ranking method, namely Multi-Objective Optimization on the basis of a Ratio Analysis plus the Full Multiplicative form (MULTIMOORA) with Pythagorean hesitant fuzzy sets. Table 1 presents the description of related works on OWPS location selection. Unfortunately, there is no IVIF-information based MCDM method for the evaluation of OWPS locations.

**Table 1:** MCDM methods for OWPS location selection.

Authors and year	MCDM method	Background
Fetanat and Khorasaninejad^35^	Hybrid ANP-DEMATEL-ELECTRE method	Fuzzy set
Wu et al.^36^	ELECTRE-III method	Intuitionistic fuzzy set
Wu et al.^37^	AHP-based model	Fuzzy set
Tercan et al.^38^	GIS-based MCDM model	Fuzzy set
Genç et al.^39^	MCDM method using GIS	Crisp set
Abdel-Basset et al.^5^	Combined MCDM method based on ANP and PROMETHEE-II	Neutrosophic set
Zhou et al.^40^	Hybrid BWM-TODIM method	Probabilistic linguistic term set
Caceoğlu et al.^41^	Integrated GIS-AHP model	Crisp set
Wang et al.^4^	AHP-WASPAS method	Spherical fuzzy set
Salvador et al.^43^	Bayesian best–worst method	Crisp set
Sánchez-Lozano et al.^42^	Integrated AHP-GIS-TOPSIS-VIKOR	Fuzzy set
Karipoğlu et al.^8^	Integrated GIS-AHP-EDAS method	Fuzzy set
Gil-García et al.^44^	Combined GIS and MCDM-based model	Crisp set
Abdel-Basset et al.^7^	Entropy-based EDAS method	Neutrosophic set
Zhou et al.^2,3^	SWARA-MULTIMOORA approach	Pythagorean hesitant fuzzy set

### Interval-valued intuitionistic fuzzy sets (IVIFSs)

Many theories and useful applications have been put forward using IVIFS context. For instance, Deveci et al.^1^ gave an incorporated decision model for assessing the renewable energy resources under IVIFS context. Kumar and Chen^25^ developed a hybrid MCDM approach with the combination of score function and the set pair analysis theory in the context of IVIFS. An improved IVIFS-based MCDM approach has presented with the consideration of DMs’ risk preference^45^. Further, they proposed their method based on TODIM and distance measure under IVIFS context. Yao and Guo^26^ proposed a new aggregation operator, means and variances for IVIFSs and presented an algorithm to solve the MCDM problems. Rathnasabapathy and Palanisami^17^ proposed a cosine similarity measure to compute the degree of similarity between IVIFSs and discussed its relevance in real-life situations. Salimian and Mousavi^18^ proposed an extended weighted distance-based approximation method and presented its application in the assessment of digital technology strategies in Covid-19 pandemic. Rani et al.^46^ studied a hybrid MULTIMOORA method for evaluating the sustainable recycling partner selection problem in small-and-medium enterprises. The proposed MULTIMOORA has combined with symmetric point of criterion and rank sum models with interval-valued intuitionistic fuzzy information. Mishra et al.^47^ developed an extended multi-attribute ideal-real comparative analysis method in the context of IVIFSs. The proposed method has applied to evaluate the wastewater treatment technologies from sustainability and uncertainty perspectives. Dağıstanlı et al.^16^ studied an improved interval-valued intuitionistic fuzzy VIKOR (VlseKriterijumska Optimizacija I Kompromisno Resenje) method for assessing the R&D projects in defense industry. By combining the group decision-making method and IVIFSs, Xhou et al.^2,3^ proposed a regional agricultural sustainability assessment method based on the water-land-energy-carbon nexus system. Further, the proposed method has implemented on a real case study in Heilongjiang Province in northeastern China to manage constrained water, energy, food and land resources. To enhance low-light color images, Jebadass and Balasubramaniam^48^ developed a new IVIFS-based enhancement technique, which offers good quality images by adjusting the contrast-limited adaptive histogram equalization system. With the use of Hamming distance, Malik and Gupta^49^ studied the division and subtraction operations over any arbitrary IVIFSs. Moreover, they presented a deterministic linear optimization technique to obtain the complete expressions for these operations. Apart from these studies, several studies have been presented in the context of IVIFSs^50–52^.

### Weighted integrated sum-product method

Using the concepts of additive ratio assessment (ARAS), WASPAS, combined compromise solution (CoCoSo) and MULTIMOORA methods, Stanujkic et al.^27^ introduced a novel MCDM approach, named as Weighted Integrated Sum Product (WISP), which uses the max normalization procedure. Zavadskas et al.^29^ presented a modified WISP model with different normalization procedures. They further emphasized the robustness of their proposed model in comparison with the classical WISP model proposed by Stanujkic et al.^53^. In addition, Zavadskas et al.^30^ proposed an intuitionistic fuzzy extension of WISP approach and employed to solve the multi-criteria contraction selection problem. Stanujkic et al.^53^ generalized the standard WISP model within the context of single-valued neutrosophic set and used to solve the contractor and industrial robot selection problems. Ulutaş et al.^54^ incorporated the WISP ranking model with MEREC criteria weighting tool and further evaluated the pallet truck selection problem. An intuitionistic fuzzy extension of WISP approach has been proposed by Hezam et al.^21,22^. Moreover, they combined the intuitionistic fuzzy WISP ranking model with double normalization procedures and closeness coefficient-based weighting model. Deveci et al.^28^ extended the classical WISP method from q-rung orthopair fuzzy information perspective and applied to a real case study on sustainable urban transportation assessment in metaverse. Till now, there is no study which combines the WISP method with distance measure, RANCOM model and IVIFS theory (Table 2).

**Table 2:** Related works on WISP method from various perspectives.

Authors and year	Method	Application
Stanujkic et al.^27^	Classical WISP method	Contractor and industrial robot selection
Zavadskas et al.^30^	Intuitionistic fuzzy WISP model	Contractor selection
Ulutaş et al.^54^	Integrated MEREC-WISP approach	Pallet truck selection
Stanujkic et al.^53^	Single valued neutrosophic WISP model	Assessment of tourist destination for nature and rural tourism
Hezam et al.^21,22^	Closeness coefficient and double normalization-based WISP method	Gerontechnology selection for aging and disabled persons
Deveci et al.^28^	SWARA-based q-rung orthopair fuzzy WISP method	Assessment of sustainable urban transportation in metaverse

In the following, we identify some issues in the existing studies:

- Several authors^31–34^ have focused their attention on the development of new distance measures for IVIFSs, but these measures generate some counter-intuitive results during the computation of degree of difference between IVIFSs.
- Few authors^21,22,27–30,53,54^ have developed the extensions of classical WISP method from crisp, intuitionistic fuzzy, single-valued neutrosophic, intuitionistic fuzzy and q-rung orthopair fuzzy perspectives, but these approaches are unable to handle the MCDM problems with interval-valued intuitionistic fuzzy information, i.e., the alternatives’ information is represented in terms of intervals rather than the exact numbers.
- Więckowski et al.^55^ proposed the Ranking Comparison (RANCOM) model to compute the subjective weight of criteria, which considers the experts’ knowledge and opinions in determining the criteria ranking order. It allows for handling the inaccuracies in expert judgments. Thus far, no one has combined the RANCOM model with WISP method in the context of interval-valued intuitionistic fuzzy information.
- Several authors^2–5,7,8,36–38^ have proposed different MCDM methods for solving OWPS location selection problem. But existing studies are not able to express the interval-valued membership and non-membership degrees in the assessment of OWPS locations. In addition, these works on OWPS location selection are failed to determine the criteria weights from objective and subjective perspectives.

To avoid the shortcomings of existing works, this study aims to develop an integrated interval-valued intuitionistic fuzzy MCDM framework to assess and prioritize the locations for offshore wind power station development. The proposed framework does not only assess the considered location alternatives through interval-valued intuitionistic fuzzy WISP method, but also computes the weights of considered criteria and decision experts during the assessment of OWPS locations. The proposed framework can help the decision experts to get more assured for ranking the OWPS locations under uncertain environment.

## Distance measure for IVIFSs

This section presents the fundamental notions related to this work and then introduces a new distance measure for IVIFSs. Comparison with existing distance measures is presented to illustrate the effectiveness of introduced measure under IVIFS environment.

### Basic concepts

As an extension of intuitionistic fuzzy set, Atanassov and Gargov^14^ suggested the concept of interval-valued intuitionistic fuzzy set to handle the uncertainty, which is mathematically defined as

#### **Definition 1**

Consider $V = \,\left\{ {\iota_{1} ,\,\iota_{2} ,\,...,\,\iota_{t} } \right\}$ be a finite universal set. In the following way, Atanassov and Gargov^14^ presented the mathematical definition of an interval-valued intuitionistic fuzzy set *P* on *V*:

1 $$P\, = \,\left\{ {\left\langle {\iota_{i} ,\,\left( {\left[ {\mu_{P}^{ - } (\iota_{i} ),\,\mu_{P}^{ + } (\iota_{i} )} \right],\,\left[ {\nu_{P}^{ - } (\iota_{i} ),\,\nu_{P}^{ + } (\iota_{i} )} \right]} \right)} \right\rangle \,:\,\iota_{i} \, \in \,V} \right\},$$

where $$0\, \le \mu_{P}^{ - } (\iota_{i} )\, \le \,\mu_{P}^{ + } (\iota_{i} )\, \le 1, \,$$$$0\, \le \nu_{P}^{ - } (\iota_{i} )\, \le \,\nu_{P}^{ + } (\iota_{i} )\, \le 1$$ and $$0\,\, \le \,\mu_{P}^{ + } \left( {\iota_{i} } \right)\, + \,\nu_{P}^{ + } \left( {\iota_{i} } \right)\, \le \,1.$$ Here, $$\mu_{P} (\iota_{i} )\, = \,\left[ {\mu_{P}^{ - } (\iota_{i} ),\,\mu_{P}^{ + } (\iota_{i} )} \right]$$ denotes the interval-valued membership degree and $$\nu_{P} (\iota_{i} )\, = \,\left[ {\nu_{P}^{ - } (\iota_{i} ),\,\nu_{P}^{ + } (\iota_{i} )} \right]$$ denote the interval-valued non-membership degree of an element $$\iota_{i}$$ in *P*, where $$\sup \left( {\mu_{P} \left( {\iota_{i} } \right)} \right)\, + \,\sup \left( {\nu_{P} \left( {\iota_{i} } \right)} \right)\, \le \,1.$$

The function $\pi_{P} \left( {\iota_{i} } \right)\, = \,\left[ {\pi_{P}^{ - } (\iota_{i} ),\,\pi_{P}^{ + } (\iota_{i} )} \right]$ defines the indeterminacy degree of an element $\iota_{i}$ to *P*, wherein $\pi_{P}^{ - } (\iota_{i} )\, = \,1\, - \,\mu_{P}^{ + } (\iota_{i} )\, - \,\nu_{P}^{ + } (\iota_{i} )$ and $\pi_{P}^{ + } (\iota_{i} )\, = \,1\, - \,\mu_{P}^{ - } (\iota_{i} )\, - \,\nu_{P}^{ - } (\iota_{i} ).$ For the simplicity, the term $\left( {\left[ {\mu_{P}^{ - } (\iota_{i} ),\,\mu_{P}^{ + } (\iota_{i} )} \right],\,\left[ {\nu_{P}^{ - } (\iota_{i} ),\,\nu_{P}^{ + } (\iota_{i} )} \right]} \right)$ is defined as the “interval-valued intuitionistic fuzzy value/number (IVIFV/IVIFN)” and symbolized by $\omega \, = \,\left( {\left[ {\mu_{\omega }^{ - } ,\,\mu_{\omega }^{ + } } \right],\,\left[ {\nu_{\omega }^{ - } ,\,\nu_{\omega }^{ + } } \right]} \right)$ which fulfills $0 \le \left( {\mu_{\omega }^{ + } } \right) + \,\,\left( {\nu_{\omega }^{ + } } \right) \le 1.$

#### **Definition 2**

Xu^15^ defined some operational laws on IVIFVs $\omega_{1} = \left( {\left[ {\mu_{1}^{ - } ,\,\mu_{1}^{ + } } \right],\,\left[ {\nu_{1}^{ - } ,\,\nu_{1}^{ + } } \right]} \right)$ and $\omega_{2} = \left( {\left[ {\mu_{2}^{ - } ,\,\mu_{2}^{ + } } \right],\,\left[ {\nu_{2}^{ - } ,\,\nu_{2}^{ + } } \right]} \right),$ presented as

- $\omega_{1} \, \subseteq \,\omega_{2}$ if and only if $\mu_{1}^{ - } \left( {\iota_{i} } \right)\, \le \,\mu_{2}^{ - } \left( {\iota_{i} } \right),\,\,\mu_{1}^{ + } \left( {\iota_{i} } \right)\, \le \,\mu_{2}^{ + } \left( {\iota_{i} } \right),\,\,\nu_{1}^{ - } \left( {\iota_{i} } \right)\, \ge \,\nu_{2}^{ - } \left( {\iota_{i} } \right)$ and $\nu_{1}^{ + } \left( {\iota_{i} } \right)\, \ge \,\nu_{2}^{ + } \left( {\iota_{i} } \right),\,\,\forall \,\iota_{i} \, \in \,V,$
- $\omega_{1} = \,\omega_{2}$ if and only if $\omega_{1} \, \subseteq \,\omega_{2}$ and $\omega_{1} \, \supseteq \,\omega_{2} ,$
- $$\omega_{1}^{c} \, = \,\left\{ {\left( {\iota_{i} ,\,\left[ {\nu_{1}^{ - } \left( {\iota_{i} } \right),\,\nu_{1}^{ + } \left( {\iota_{i} } \right)} \right],\,\left[ {\mu_{1}^{ - } \left( {\iota_{i} } \right),\,\mu_{1}^{ + } \left( {\iota_{i} } \right)} \right]} \right)\,{|}\,\iota_{i} \, \in \,V} \right\},$$
- $$\omega_{1} \cup \,\omega_{2} \, = \,\left\{ {\left( \begin{gathered} \iota_{i} ,\,\,\left[ {\mu_{1}^{ - } \left( {\iota_{i} } \right)\, \vee \mu_{2}^{ - } \left( {\iota_{i} } \right),\,\mu_{1}^{ + } \left( {\iota_{i} } \right)\, \vee \,\mu_{2}^{ + } \left( {\iota_{i} } \right)} \right], \hfill \\ \,\,\,\,\,\,\left[ {\nu_{1}^{ - } \left( {\iota_{i} } \right)\, \wedge \nu_{2}^{ - } \left( {\iota_{i} } \right),\,\nu_{1}^{ + } \left( {\iota_{i} } \right)\, \wedge \,\,\nu_{2}^{ + } \left( {\iota_{i} } \right)} \right] \hfill \\ \end{gathered} \right)\,\left| {\,\iota_{i} \, \in \,V} \right.} \right\}$$
- $$\omega_{1} \cap \,\omega_{2} \, = \,\left\{ {\left( \begin{gathered} \iota_{i} ,\,\,\left[ {\mu_{1}^{ - } \left( {\iota_{i} } \right)\, \wedge \,\mu_{2}^{ - } \left( {\iota_{i} } \right),\,\mu_{1}^{ + } \left( {\iota_{i} } \right)\, \wedge \,\mu_{2}^{ + } \left( {\iota_{i} } \right)} \right], \hfill \\ \,\,\,\,\,\,\left[ {\nu_{1}^{ - } \left( {\iota_{i} } \right)\, \vee \,\nu_{2}^{ - } \left( {\iota_{i} } \right),\,\,\nu_{1}^{ + } \left( {\iota_{i} } \right)\, \vee \,\nu_{2}^{ + } \left( {\iota_{i} } \right)} \right] \hfill \\ \end{gathered} \right)\,|\,\iota_{i} \, \in \,V} \right\}.$$

#### **Definition 3**

For any IVIFN $\omega \, = \,\left( {\left[ {\mu_{\omega }^{ - } ,\,\mu_{\omega }^{ + } } \right],\,\left[ {\nu_{\omega }^{ - } ,\,\nu_{\omega }^{ + } } \right]} \right),$ Xu et al.^56^ defined the score and accuracy functions, given by Eq. (2) and Eq. (3), respectively.

2 $${\mathbb{S}}\left( \omega \right) = \frac{1}{2}\left( {\frac{1}{2}\left( {\mu_{\omega }^{ - } + \,\mu_{\omega }^{ + } - \nu_{\omega }^{ - } - \nu_{\omega }^{ + } } \right) + 1} \right),$$

3 $${\mathbb{H}}\left( \omega \right) = \frac{1}{2}\left( {\mu_{\omega }^{ - } + \,\mu_{\omega }^{ + } + \,\nu_{\omega }^{ - } + \,\nu_{\omega }^{ + } } \right).$$

#### **Definition 4**

For a set of IVIFNs $\omega = \left\{ {\omega_{1} ,\,\omega_{2} ,...,\,\omega_{t} } \right\},$ where $\omega_{k} = \left( {\left[ {\mu_{k}^{ - } ,\,\mu_{k}^{ + } } \right],\left[ {\nu_{k}^{ - } ,\,\nu_{k}^{ + } } \right]} \right),\,k = 1,2, \cdots ,t,$ Xu^15^ defined the interval-valued intuitionistic fuzzy weighted averaging and geometric operators, given as

4 $$\mathop \oplus \limits_{k = 1}^{t} \alpha_{k} \,\omega_{k} = \left( {\left[ {1 - \prod\limits_{k = 1}^{t} {\left( {1 - \mu_{k}^{ - } } \right)^{{\alpha_{k} }} } ,\,\,1 - \prod\limits_{k = 1}^{t} {\left( {1 - \mu_{k}^{ + } } \right)^{{\alpha_{k} }} } } \right],\left[ {\prod\limits_{k = 1}^{t} {\left( {\nu_{k}^{ - } } \right)^{{\alpha_{k} }} } ,\,\,\prod\limits_{k = 1}^{t} {\left( {\nu_{k}^{ + } } \right)^{{\alpha_{k} }} } } \right]} \right),$$

5 $$\mathop \otimes \limits_{k = 1}^{t} \alpha_{k} \,\omega_{k} = \left( {\left[ {\prod\limits_{k = 1}^{t} {\left( {\mu_{k}^{ - } } \right)^{{\alpha_{k} }} } ,\,\,\prod\limits_{k = 1}^{t} {\left( {\mu_{k}^{ + } } \right)^{{\alpha_{k} }} } } \right],\,\left[ {1 - \prod\limits_{k = 1}^{t} {\left( {1 - \nu_{k}^{ - } } \right)^{{\alpha_{k} }} } ,\,\,1 - \prod\limits_{k = 1}^{t} {\left( {1 - \nu_{k}^{ + } } \right)^{{\alpha_{k} }} } } \right]} \right).$$

#### Definition 5

^34^ Let $P,\,Q\, \in \,IVIFSs\left( V \right).$ An interval-valued intuitionistic fuzzy distance measure $d\,:IVIFSs\left( V \right) \times IVIFSs\left( V \right) \to [0,\,1]$ is a real-valued function which holds the following requirements:

- (C1). $$0 \le d\left( {P,\,Q} \right) \le 1,$$
- (C2). $$d\left( {P,\,Q} \right) = d\left( {Q,\,P} \right),$$
- (C3). $$d\left( {P,\,Q} \right) = 0 \Leftrightarrow P\, = \,Q,$$
- (C4). If $$P\, \subseteq \,Q \subseteq \,R,$$ then $$d\left( {P,\,R} \right) \ge d\left( {P,\,Q} \right)$$ and $$d\left( {P,\,R} \right) \ge d\left( {Q,\,R} \right),$$$$\forall \,\,P,\,Q,\,R\,\, \in IVIFSs\left( V \right).$$

### New distance measure for IVIFSs

Let *P* and *Q* be two IVIFSs. To quantify the difference between IVIFSs *P* and *Q*, we define a novel interval-valued intuitionistic fuzzy distance measure, presented as

6 $$d\left( {P,\,Q} \right) = \,\frac{1}{2n}\,\sum\limits_{i\, = 1}^{n} {\left[ {g\left( {\left| {\mu_{1}^{ - } (\iota_{i} ) - \,\mu_{2}^{ - } (\iota_{i} )} \right|,\,\left| {\nu_{1}^{ - } (\iota_{i} ) - \,\nu_{2}^{ - } (\iota_{i} )} \right|} \right)\, + \,h\left( {\left| {\mu_{1}^{ + } (\iota_{i} ) - \,\mu_{2}^{ + } (\iota_{i} )} \right|,\,\left| {\nu_{1}^{ + } (\iota_{i} ) - \,\nu_{2}^{ + } (\iota_{i} )} \right|} \right)} \right]},$$

where $^{\prime}g^{\prime}$ and $^{\prime}h^{\prime}$ are *t*-conorms.

#### **Theorem 1**

*The real-valued function given by Eq. *(6)* is a valid distance measure for IVIFSs.*

#### *Proof*

(C1)-(C2). By definition and operational laws of IVIFSs, the proofs are obvious.

(C3). If *P* = *Q*, then it is obvious from Eq. (6) that $d\left( {P,\,Q} \right)\, = \,0.$ Conversely, if $d\left( {P,\,Q} \right)\, = \,0,$ then from Eq. (6), we have

$$\frac{1}{2n}\,\sum\limits_{i\, = 1}^{n} {\left[ {g\left( {\left| {\mu_{1}^{ - } (\iota_{i} ) - \,\mu_{2}^{ - } (\iota_{i} )} \right|,\,\left| {\nu_{1}^{ - } (\iota_{i} ) - \,\nu_{2}^{ - } (\iota_{i} )} \right|} \right)\, + \,h\left( {\left| {\mu_{1}^{ + } (\iota_{i} ) - \,\mu_{2}^{ + } (\iota_{i} )} \right|,\,\left| {\nu_{1}^{ + } (\iota_{i} ) - \,\nu_{2}^{ + } (\iota_{i} )} \right|} \right)} \right]} \, = \,0,\,\,\forall \,\iota_{i} \, \in \,V,$$

$$\Rightarrow g\left( {\left| {\mu_{1}^{ - } (\iota_{i} ) - \,\mu_{2}^{ - } (\iota_{i} )} \right|,\,\left| {\nu_{1}^{ - } (\iota_{i} ) - \,\nu_{2}^{ - } (\iota_{i} )} \right|} \right)\, + \,h\left( {\left| {\mu_{1}^{ + } (\iota_{i} ) - \,\mu_{2}^{ + } (\iota_{i} )} \right|,\,\left| {\nu_{1}^{ + } (\iota_{i} ) - \,\nu_{2}^{ + } (\iota_{i} )} \right|} \right)\, = \,0,\,\,\forall \,\iota_{i} \, \in \,V,$$

$$\Rightarrow g\left( {\left| {\mu_{1}^{ - } (\iota_{i} ) - \,\mu_{2}^{ - } (\iota_{i} )} \right|,\,\left| {\nu_{1}^{ - } (\iota_{i} ) - \,\nu_{2}^{ - } (\iota_{i} )} \right|} \right)\, = \,0\;\;{\text{and}}\;\;h\left( {\left| {\mu_{1}^{ + } (\iota_{i} ) - \,\mu_{2}^{ + } (\iota_{i} )} \right|,\,\left| {\nu_{1}^{ + } (\iota_{i} ) - \,\nu_{2}^{ + } (\iota_{i} )} \right|} \right)\, = \,0,\,\forall \,\iota_{i} \, \in \,V,$$

$$\Rightarrow \,\left| {\mu_{1}^{ - } (\iota_{i} ) - \,\mu_{2}^{ - } (\iota_{i} )} \right|\, = \,0,\;\;\left| {\nu_{1}^{ - } (\iota_{i} ) - \,\nu_{2}^{ - } (\iota_{i} )} \right|\, = \,0,\;\left| {\mu_{1}^{ + } (\iota_{i} ) - \,\mu_{2}^{ + } (\iota_{i} )} \right|\, = \,0\;{\text{and}}\;\left| {\nu_{1}^{ + } (\iota_{i} ) - \,\nu_{2}^{ + } (\iota_{i} )} \right|\, = \,0,\,\forall \,\iota_{i} \, \in \,V,$$

$$\Rightarrow \,\mu_{1}^{ - } (\iota_{i} ) = \,\mu_{2}^{ - } (\iota_{i} ),\;\nu_{1}^{ - } (\iota_{i} ) = \,\,\nu_{2}^{ - } (\iota_{i} ),\;\mu_{1}^{ + } (\iota_{i} ) = \,\mu_{2}^{ + } (\iota_{i} )\;{\text{and}}\;\nu_{1}^{ + } (\iota_{i} ) = \nu_{2}^{ + } (\iota_{i} ),\,\forall \,\iota_{i} \, \in \,V,$$

$$\Rightarrow \,\left\langle {\left[ {\mu_{1}^{ - } (\iota_{i} ),\,\mu_{1}^{ + } (\iota_{i} )} \right],\,\left[ {\nu_{1}^{ - } (\iota_{i} ),\,\nu_{1}^{ + } (\iota_{i} )} \right]} \right\rangle = \left\langle {\left[ {\mu_{2}^{ - } (\iota_{i} ),\,\mu_{2}^{ + } (\iota_{i} )} \right],\,\left[ {\nu_{2}^{ - } (\iota_{i} ),\,\nu_{2}^{ + } (\iota_{i} )} \right]} \right\rangle ,\,\,\forall \,\iota_{i} \in V,$$

$$\Rightarrow \,P\, = \,Q.$$

(C4). Let $$P,\,Q,\,R\, \in \,IVIFSs\left( V \right),$$ where $$P\, = \,\left\{ {\left\langle {\iota_{i} ,\,\left( {\left[ {\mu_{1}^{ - } (\iota_{i} ),\,\mu_{1}^{ + } (\iota_{i} )} \right],\,\left[ {\nu_{1}^{ - } (\iota_{i} ),\,\nu_{1}^{ + } (\iota_{i} )} \right]} \right)} \right\rangle \,:\,\iota_{i} \, \in \,V} \right\},$$ $$Q\, = \,\left\{ {\left\langle {\iota_{i} ,\,\left( {\left[ {\mu_{2}^{ - } (\iota_{i} ),\,\mu_{2}^{ + } (\iota_{i} )} \right],\,\left[ {\nu_{2}^{ - } (\iota_{i} ),\,\nu_{2}^{ + } (\iota_{i} )} \right]} \right)} \right\rangle \,:\,\iota_{i} \, \in \,V} \right\}$$ and $$R\, = \,\left\{ {\left\langle {\iota_{i} ,\,\left( {\left[ {\mu_{3}^{ - } (\iota_{i} ),\,\mu_{3}^{ + } (\iota_{i} )} \right],\,\left[ {\nu_{3}^{ - } (\iota_{i} ),\,\nu_{3}^{ + } (\iota_{i} )} \right]} \right)} \right\rangle \,:\,\iota_{i} \, \in \,V} \right\}.$$ If $$P\, \subseteq \,Q \subseteq \,R,$$ then $$\mu_{1}^{ - } (\iota_{i} )\,\, \le \,\mu_{2}^{ - } (\iota_{i} )\, \le \,\mu_{3}^{ - } (\iota_{i} ),$$$$\mu_{1}^{ + } (\iota_{i} )\,\, \le \,\mu_{2}^{ + } (\iota_{i} )\, \le \,\mu_{3}^{ + } (\iota_{i} ),$$$$\nu_{3}^{ - } (\iota_{i} )\,\, \le \,\nu_{2}^{ - } (\iota_{i} )\, \le \,\nu_{1}^{ - } (\iota_{i} )$$ and $$\nu_{3}^{ + } (\iota_{i} )\,\, \le \,\nu_{2}^{ + } (\iota_{i} )\, \le \,\nu_{1}^{ + } (\iota_{i} ),\,\,\forall \,\iota_{i} \, \in \,V.$$ Therefore, $$\left| {\mu_{1}^{ - } (\iota_{i} ) - \,\mu_{2}^{ - } (\iota_{i} )} \right|$$ $$\le \,\left| {\mu_{1}^{ - } (\iota_{i} ) - \,\mu_{3}^{ - } (\iota_{i} )} \right|,$$ $$\left| {\mu_{1}^{ + } (\iota_{i} ) - \,\mu_{2}^{ + } (\iota_{i} )} \right|\,$$ $$\le \,\left| {\mu_{1}^{ + } (\iota_{i} ) - \,\mu_{3}^{ + } (\iota_{i} )} \right|,$$ $$\left| {\nu_{1}^{ - } (\iota_{i} ) - \,\nu_{2}^{ - } (\iota_{i} )} \right|$$ $$\, \le \,\left| {\nu_{1}^{ - } (\iota_{i} ) - \,\nu_{3}^{ - } (\iota_{i} )} \right|$$ and $$\left| {\nu_{1}^{ + } (\iota_{i} ) - \,\nu_{2}^{ + } (\iota_{i} )} \right|$$ $$\, \le \,\left| {\nu_{1}^{ + } (\iota_{i} ) - \,\nu_{3}^{ + } (\iota_{i} )} \right|,\,\forall \,\iota_{i} \, \in \,V.$$ Also, $$\left| {\mu_{2}^{ - } (\iota_{i} ) - \,\mu_{3}^{ - } (\iota_{i} )} \right|\,$$$$\le \,\left| {\mu_{1}^{ - } (\iota_{i} ) - \,\mu_{3}^{ - } (\iota_{i} )} \right|,$$$$\left| {\mu_{2}^{ + } (\iota_{i} ) - \,\mu_{3}^{ + } (\iota_{i} )} \right|$$$$\le \,\left| {\mu_{1}^{ + } (\iota_{i} ) - \,\mu_{3}^{ + } (\iota_{i} )} \right|,$$$$\left| {\nu_{2}^{ - } (\iota_{i} ) - \,\nu_{3}^{ - } (\iota_{i} )} \right|\, \le \,\left| {\nu_{1}^{ - } (\iota_{i} ) - \,\nu_{3}^{ - } (\iota_{i} )} \right|$$ and $$\left| {\nu_{2}^{ + } (\iota_{i} ) - \,\nu_{3}^{ + } (\iota_{i} )} \right|\, \le$$$$\left| {\nu_{1}^{ + } (\iota_{i} ) - \,\nu_{3}^{ + } (\iota_{i} )} \right|,\,\forall \,\iota_{i} \, \in \,V.$$

It implies that

$$\begin{gathered} g\left( {\left| {\mu_{1}^{ - } (\iota_{i} ) - \,\mu_{2}^{ - } (\iota_{i} )} \right|,\,\left| {\nu_{1}^{ - } (\iota_{i} ) - \,\nu_{2}^{ - } (\iota_{i} )} \right|} \right)\, + \,h\left( {\left| {\mu_{1}^{ + } (\iota_{i} ) - \,\mu_{2}^{ + } (\iota_{i} )} \right|,\,\left| {\nu_{1}^{ + } (\iota_{i} ) - \,\nu_{2}^{ + } (\iota_{i} )} \right|} \right) \hfill \\ \le \,g\left( {\left| {\mu_{1}^{ - } (\iota_{i} ) - \,\mu_{3}^{ - } (\iota_{i} )} \right|,\,\left| {\nu_{1}^{ - } (\iota_{i} ) - \,\nu_{3}^{ - } (\iota_{i} )} \right|} \right)\, + \,h\left( {\left| {\mu_{1}^{ + } (\iota_{i} ) - \,\mu_{3}^{ + } (\iota_{i} )} \right|,\,\left| {\nu_{1}^{ + } (\iota_{i} ) - \,\nu_{3}^{ + } (\iota_{i} )} \right|} \right) \hfill \\ \end{gathered}$$

and

$$\begin{gathered} g\left( {\left| {\mu_{2}^{ - } (\iota_{i} ) - \,\mu_{3}^{ - } (\iota_{i} )} \right|,\,\left| {\nu_{2}^{ - } (\iota_{i} ) - \,\nu_{3}^{ - } (\iota_{i} )} \right|} \right)\, + \,h\left( {\left| {\mu_{2}^{ + } (\iota_{i} ) - \,\mu_{3}^{ + } (\iota_{i} )} \right|,\,\left| {\nu_{2}^{ + } (\iota_{i} ) - \,\nu_{3}^{ + } (\iota_{i} )} \right|} \right) \hfill \\ \le \,g\left( {\left| {\mu_{1}^{ - } (\iota_{i} ) - \,\mu_{3}^{ - } (\iota_{i} )} \right|,\,\left| {\nu_{1}^{ - } (\iota_{i} ) - \,\nu_{3}^{ - } (\iota_{i} )} \right|} \right)\, + \,h\left( {\left| {\mu_{1}^{ + } (\iota_{i} ) - \,\mu_{3}^{ + } (\iota_{i} )} \right|,\,\left| {\nu_{1}^{ + } (\iota_{i} ) - \,\nu_{3}^{ + } (\iota_{i} )} \right|} \right),\,\,\forall \,\iota_{i} \, \in \,V. \hfill \\ \end{gathered}$$

It implies that $$d\left( {P,\,R} \right) \ge d\left( {P,\,Q} \right)$$ and $$d\left( {P,\,R} \right) \ge d\left( {Q,\,R} \right),$$$$\forall \,\,P,\,Q,\,R\,\, \in IVIFSs\left( V \right).$$

*Note*

1. If the *t*-conorm is $\min \left\{ {1,\,a + \,b} \right\},$ then
  7 $$d_{1} \left( {P,\,Q} \right)\, = \,\frac{1}{2n}\,\sum\limits_{i\, = 1}^{n} {\left( {\min \left( {1,\,\left| {\mu_{1}^{ - } (\iota_{i} ) - \,\mu_{2}^{ - } (\iota_{i} )} \right|\, + \,\left| {\nu_{1}^{ - } (\iota_{i} ) - \,\nu_{2}^{ - } (\iota_{i} )} \right|} \right)\, + \min \left( {1,\,\left| {\mu_{1}^{ + } (\iota_{i} ) - \,\mu_{2}^{ + } (\iota_{i} )} \right|\, + \,\left| {\nu_{1}^{ + } (\iota_{i} ) - \,\nu_{2}^{ + } (\iota_{i} )} \right|} \right)} \right)} \,.$$
2. If the *t*-conorm is $a\, + \,b\, - \,a.b,$ then
  8 $$d_{2} \left( {P,\,Q} \right)\, = \,\,\frac{1}{n}\,\sum\limits_{i\, = 1}^{n} {\left[ \begin{gathered} \left| {\mu_{1}^{ - } (\iota_{i} ) - \,\mu_{2}^{ - } (\iota_{i} )} \right|\, + \,\left| {\nu_{1}^{ - } (\iota_{i} ) - \,\nu_{2}^{ - } (\iota_{i} )} \right| + \left| {\mu_{1}^{ + } (\iota_{i} ) - \,\mu_{2}^{ + } (\iota_{i} )} \right| + \,\left| {\nu_{1}^{ + } (\iota_{i} ) - \,\nu_{2}^{ + } (\iota_{i} )} \right| \hfill \\ - \,\left| {\mu_{1}^{ - } (\iota_{i} ) - \,\mu_{2}^{ - } (\iota_{i} )} \right|\,.\,\left| {\nu_{1}^{ - } (\iota_{i} ) - \,\nu_{2}^{ - } (\iota_{i} )} \right| - \,\left| {\mu_{1}^{ + } (\iota_{i} ) - \,\mu_{2}^{ + } (\iota_{i} )} \right|.\,\left| {\nu_{1}^{ + } (\iota_{i} ) - \,\nu_{2}^{ + } (\iota_{i} )} \right| \hfill \\ \end{gathered} \right]} .$$

### Comparative analysis

In this part of study, we compare the proposed and existing interval-valued intuitionistic fuzzy distance measures. For this purpose, we firstly recall some of the well-known distance measures given by Xu and Chen^34^, Ming-Mei et al.^31^, Tiwari and Gupta^33^ and Rashid et al.^32^.

Normalized Hamming distance measure^34^

9 $$d_{NH} \left( {P,\,Q} \right)\, = \,\,\frac{1}{4n}\,\sum\limits_{i\, = 1}^{n} {\left( {\left| {\mu_{1}^{ - } (\iota_{i} ) - \,\mu_{2}^{ - } (\iota_{i} )} \right| + \,\left| {\mu_{1}^{ + } (\iota_{i} ) - \,\mu_{2}^{ + } (\iota_{i} )} \right| + \,\left| {\nu_{1}^{ - } (\iota_{i} ) - \,\nu_{2}^{ - } (\iota_{i} )} \right| + \,\left| {\nu_{1}^{ + } (\iota_{i} ) - \,\nu_{2}^{ + } (\iota_{i} )} \right|} \right)} .$$

Normalized Euclidean distance measure^34^

10 $$d_{NE} \left( {P,\,Q} \right)\, = \sqrt {\frac{1}{4n}\,\sum\limits_{i\, = 1}^{n} {\left( {\left( {\mu_{1}^{ - } (\iota_{i} ) - \,\mu_{2}^{ - } (\iota_{i} )} \right)^{2} + \,\left( {\mu_{1}^{ + } (\iota_{i} ) - \,\mu_{2}^{ + } (\iota_{i} )} \right)^{2} + \,\left( {\nu_{1}^{ - } (\iota_{i} ) - \,\nu_{2}^{ - } (\iota_{i} )} \right)^{2} + \,\left( {\nu_{1}^{ + } (\iota_{i} ) - \,\nu_{2}^{ + } (\iota_{i} )} \right)^{2} } \right)} } .$$

Normalized Hamming distance measure based on Hausdorff metric^34^

11 $$d_{NH - HM} \left( {P,\,Q} \right)\, = \,\,\frac{1}{n}\,\sum\limits_{i\, = 1}^{n} {\max \left\{ {\left| {\mu_{1}^{ - } (\iota_{i} ) - \,\mu_{2}^{ - } (\iota_{i} )} \right|,\,\,\left| {\mu_{1}^{ + } (\iota_{i} ) - \,\mu_{2}^{ + } (\iota_{i} )} \right|,\,\,\left| {\nu_{1}^{ - } (\iota_{i} ) - \,\nu_{2}^{ - } (\iota_{i} )} \right|,\,\,\left| {\nu_{1}^{ + } (\iota_{i} ) - \,\nu_{2}^{ + } (\iota_{i} )} \right|} \right\}}.$$

Normalized Euclidean distance measure based on Hausdorff metric^34^

12 $$d_{NE - HM} \left( {P,\,Q} \right)\, = \,\sqrt {\frac{1}{n}\,\sum\limits_{i\, = 1}^{n} {\max } \left\{ {\left( {\mu_{1}^{ - } (\iota_{i} ) - \,\mu_{2}^{ - } (\iota_{i} )} \right)^{2} ,\,\left( {\mu_{1}^{ + } (\iota_{i} ) - \,\mu_{2}^{ + } (\iota_{i} )} \right)^{2} ,\,\left( {\nu_{1}^{ - } (\iota_{i} ) - \,\nu_{2}^{ - } (\iota_{i} )} \right)^{2} ,\,\left( {\nu_{1}^{ + } (\iota_{i} ) - \,\nu_{2}^{ + } (\iota_{i} )} \right)^{2} } \right\}} .$$

Distance measure by Ming et al.^31^

13 $$d_{M} \left( {P,\,Q} \right)\, = \,\sqrt {\frac{1}{4\,n}\,\sum\limits_{i\, = 1}^{n} {\left\{ \begin{gathered} \left( {\mu_{1}^{ - } (\iota_{i} ) - \,\mu_{2}^{ - } (\iota_{i} )} \right)^{2} + \,\left( {\mu_{1}^{ + } (\iota_{i} ) - \,\mu_{2}^{ + } (\iota_{i} )} \right)^{2} + \,\left( {\nu_{1}^{ - } (\iota_{i} ) - \,\nu_{2}^{ - } (\iota_{i} )} \right)^{2} \hfill \\ + \,\left( {\nu_{1}^{ + } (\iota_{i} ) - \,\nu_{2}^{ + } (\iota_{i} )} \right)^{2} + \,\left( {\pi_{1}^{ - } (\iota_{i} ) - \,\pi_{2}^{ - } (\iota_{i} )} \right)^{2} + \,\left( {\pi_{1}^{ + } (\iota_{i} ) - \,\pi_{2}^{ + } (\iota_{i} )} \right)^{2} \hfill \\ \end{gathered} \right\}} } .$$

Distance measure by Tiwari and Gupta^33^

14 $$d_{TG1} \left( {P,\,Q} \right)\, = \,\,\frac{1}{8n}\,\sum\limits_{i\, = 1}^{n} {\left( \begin{gathered} \left| {\mu_{1}^{ - } (\iota_{i} ) - \,\mu_{2}^{ - } (\iota_{i} )} \right| + \,\left| {\mu_{1}^{ + } (\iota_{i} ) - \,\mu_{2}^{ + } (\iota_{i} )} \right| + \,\left| {\nu_{1}^{ - } (\iota_{i} ) - \,\nu_{2}^{ - } (\iota_{i} )} \right| \hfill \\ + \,\left| {\nu_{1}^{ + } (\iota_{i} ) - \,\nu_{2}^{ + } (\iota_{i} )} \right|\, + \,\left| {\pi_{1}^{ - } (\iota_{i} ) - \,\pi_{2}^{ - } (\iota_{i} )} \right| + \,\left| {\pi_{1}^{ + } (\iota_{i} ) - \,\pi_{2}^{ + } (\iota_{i} )} \right| \hfill \\ \end{gathered} \right)} .$$

15 $$d_{TG2} \left( {P,\,Q} \right)\, = \,\sqrt {\frac{1}{12n}\,\sum\limits_{i\, = 1}^{n} {\left( \begin{gathered} \left( {\mu_{1}^{ - } (\iota_{i} ) - \,\mu_{2}^{ - } (\iota_{i} )} \right)^{2} + \,\left( {\mu_{1}^{ + } (\iota_{i} ) - \,\mu_{2}^{ + } (\iota_{i} )} \right)^{2} + \,\left( {\nu_{1}^{ - } (\iota_{i} ) - \,\nu_{2}^{ - } (\iota_{i} )} \right)^{2} \hfill \\ + \,\left( {\nu_{1}^{ + } (\iota_{i} ) - \,\nu_{2}^{ + } (\iota_{i} )} \right)^{2} + \,\left( {\pi_{1}^{ - } (\iota_{i} ) - \,\pi_{2}^{ - } (\iota_{i} )} \right)^{2} + \,\left( {\pi_{1}^{ + } (\iota_{i} ) - \,\pi_{2}^{ + } (\iota_{i} )} \right)^{2} \hfill \\ \end{gathered} \right)} } .$$

16 $$d_{TG3} \left( {P,\,Q} \right)\, = \,\,\frac{1}{4\,n}\,\sum\limits_{i\, = 1}^{n} {\left\{ \begin{gathered} \left| {\mu_{1}^{ - } (\iota_{i} ) - \,\mu_{2}^{ - } (\iota_{i} )} \right| \vee \,\,\left| {\mu_{1}^{ + } (\iota_{i} ) - \,\mu_{2}^{ + } (\iota_{i} )} \right| \hfill \\ + \,\left| {\nu_{1}^{ - } (\iota_{i} ) - \,\nu_{2}^{ - } (\iota_{i} )} \right| \vee \,\,\left| {\nu_{1}^{ + } (\iota_{i} ) - \,\nu_{2}^{ + } (\iota_{i} )} \right| \hfill \\ + \,\left| {\pi_{1}^{ - } (\iota_{i} ) - \,\pi_{2}^{ - } (\iota_{i} )} \right| \vee \,\,\left| {\pi_{1}^{ + } (\iota_{i} ) - \,\pi_{2}^{ + } (\iota_{i} )} \right| \hfill \\ \end{gathered} \right\}} .$$

17 $$d_{TG4} \left( {P,\,Q} \right)\, = \,\,\frac{1}{4\,n}\,\sum\limits_{i\, = 1}^{n} {\max \left\{ \begin{gathered} \frac{{\left| {\mu_{1}^{ - } (\iota_{i} ) - \,\mu_{2}^{ - } (\iota_{i} )} \right| + \,\left| {\mu_{1}^{ + } (\iota_{i} ) - \,\mu_{2}^{ + } (\iota_{i} )} \right|}}{2}, \hfill \\ \frac{{\left| {\nu_{1}^{ - } (\iota_{i} ) - \,\nu_{2}^{ - } (\iota_{i} )} \right| + \,\,\left| {\nu_{1}^{ + } (\iota_{i} ) - \,\nu_{2}^{ + } (\iota_{i} )} \right|}}{2}, \hfill \\ \frac{{\left| {\pi_{1}^{ - } (\iota_{i} ) - \,\pi_{2}^{ - } (\iota_{i} )} \right| + \,\,\left| {\pi_{1}^{ + } (\iota_{i} ) - \,\pi_{2}^{ + } (\iota_{i} )} \right|}}{2} \hfill \\ \end{gathered} \right\}} .$$

18 $$d_{TG5} \left( {P,\,Q} \right)\, = \,\,\frac{1}{2\,n}\,\sum\limits_{i\, = 1}^{n} {\left\{ \begin{gathered} \frac{\begin{gathered} \left| {\mu_{1}^{ - } (\iota_{i} ) - \,\mu_{2}^{ - } (\iota_{i} )} \right| + \,\left| {\mu_{1}^{ + } (\iota_{i} ) - \,\mu_{2}^{ + } (\iota_{i} )} \right| + \left| {\nu_{1}^{ - } (\iota_{i} ) - \,\nu_{2}^{ - } (\iota_{i} )} \right| \hfill \\ + \,\left| {\nu_{1}^{ + } (\iota_{i} ) - \,\nu_{2}^{ + } (\iota_{i} )} \right|\, + \,\left| {\pi_{1}^{ - } (\iota_{i} ) - \,\pi_{2}^{ - } (\iota_{i} )} \right| + \,\left| {\pi_{1}^{ + } (\iota_{i} ) - \,\pi_{2}^{ + } (\iota_{i} )} \right| \hfill \\ \end{gathered} }{8} \hfill \\ + \max \left\{ {\frac{\begin{gathered} \left| {\mu_{1}^{ - } (\iota_{i} ) - \,\mu_{2}^{ - } (\iota_{i} )} \right| + \,\left| {\mu_{1}^{ + } (\iota_{i} ) - \,\mu_{2}^{ + } (\iota_{i} )} \right|, \hfill \\ \left| {\nu_{1}^{ - } (\iota_{i} ) - \,\nu_{2}^{ - } (\iota_{i} )} \right| + \,\,\left| {\nu_{1}^{ + } (\iota_{i} ) - \,\nu_{2}^{ + } (\iota_{i} )} \right|, \hfill \\ \left| {\pi_{1}^{ - } (\iota_{i} ) - \,\pi_{2}^{ - } (\iota_{i} )} \right| + \,\,\left| {\pi_{1}^{ + } (\iota_{i} ) - \,\pi_{2}^{ + } (\iota_{i} )} \right| \hfill \\ \end{gathered} }{4}} \right\} \hfill \\ \end{gathered} \right\}} .$$

19 $$d_{TG6} \left( {P,\,Q} \right)\, = \,\,\left\{ {\frac{1}{12\,n}\,\sum\limits_{i\, = 1}^{n} {\left( \begin{gathered} \left( {\left| {\mu_{1}^{ - } (\iota_{i} ) - \,\mu_{2}^{ - } (\iota_{i} )} \right| \vee \,\,\left| {\mu_{1}^{ + } (\iota_{i} ) - \,\mu_{2}^{ + } (\iota_{i} )} \right|} \right)^{p} \hfill \\ + \,\left( {\left| {\nu_{1}^{ - } (\iota_{i} ) - \,\nu_{2}^{ - } (\iota_{i} )} \right| \vee \,\,\left| {\nu_{1}^{ + } (\iota_{i} ) - \,\nu_{2}^{ + } (\iota_{i} )} \right|} \right)^{p} \hfill \\ + \,\left( {\left| {\pi_{1}^{ - } (\iota_{i} ) - \,\pi_{2}^{ - } (\iota_{i} )} \right| \vee \,\,\left| {\pi_{1}^{ + } (\iota_{i} ) - \,\pi_{2}^{ + } (\iota_{i} )} \right|} \right)^{p} \hfill \\ \end{gathered} \right)} } \right\}^{1/p} .$$

Distance measure by Rashid et al.^32^

20 $$d_{R} \left( {P,\,Q} \right)\, = \,\,\frac{1}{2}\,\left( \begin{gathered} \sum\limits_{i\, = 1}^{n} {\left( {\min \left\{ {\left| {\mu_{1}^{ - } (\iota_{i} ) - \,\mu_{2}^{ - } (\iota_{i} )} \right|,\,\,\left| {\mu_{1}^{ + } (\iota_{i} ) - \,\mu_{2}^{ + } (\iota_{i} )} \right|,\,\,\left| {\nu_{1}^{ - } (\iota_{i} ) - \,\nu_{2}^{ - } (\iota_{i} )} \right|,\,\,\left| {\nu_{1}^{ + } (\iota_{i} ) - \,\nu_{2}^{ + } (\iota_{i} )} \right|} \right\}} \right)} \hfill \\ + \sum\limits_{i\, = 1}^{n} {\left( {\max \left\{ {\left| {\mu_{1}^{ - } (\iota_{i} ) - \,\mu_{2}^{ - } (\iota_{i} )} \right|,\,\,\left| {\mu_{1}^{ + } (\iota_{i} ) - \,\mu_{2}^{ + } (\iota_{i} )} \right|,\,\,\left| {\nu_{1}^{ - } (\iota_{i} ) - \,\nu_{2}^{ - } (\iota_{i} )} \right|,\,\,\left| {\nu_{1}^{ + } (\iota_{i} ) - \,\nu_{2}^{ + } (\iota_{i} )} \right|} \right\}} \right)} \hfill \\ \end{gathered} \right)\,.$$

To compare the proposed and existing distance measures, we took some examples in Table 3. On the basis of Table 3, we present the following interesting points:

- For two different IVIFSs given by Set 2 and Set 3, existing distance measures $d_{NH} \left( {P,\,Q} \right),$ $d_{TG1} \left( {P,\,Q} \right),$ $d_{TG3} \left( {P,\,Q} \right),$ $d_{TG4} \left( {P,\,Q} \right),$ $d_{TG5} \left( {P,\,Q} \right)$ and $d_{R} \left( {P,\,Q} \right)$ generate counter-intuitive results.
- For Set 1 and Set 4, we can see that all of the distance measures evaluate the difference between IVIFSs *P* and *Q* very well.
- When compared the distance measure outcomes on the Set 2 and Set 5, we obtain that the distance measures $d_{NH - HM} \left( {P,\,Q} \right),$ $d_{NE - HM} \left( {P,\,Q} \right),$ $d_{M} \left( {P,\,Q} \right),$ $d_{TG1} \left( {P,\,Q} \right),$ $d_{TG2} \left( {P,\,Q} \right),$ $d_{TG3} \left( {P,\,Q} \right),$ $d_{TG4} \left( {P,\,Q} \right),$ $d_{TG5} \left( {P,\,Q} \right)$ and $d_{1} \left( {P,\,Q} \right)$ present the counter-intuitive cases.
- Next, when compared the distance measure $d_{2} \left( {P,\,Q} \right)$ under Set 2 and Set 3 or Set 2 and Set 5, we can see that the proposed interval-valued intuitionistic fuzzy distance measure describes the discrimination degree better than other existing measures.

**Table 3:** Comparative results obtained by the proposed and existing measures (bold type represents the counter-intuitive cases).

IVIFSs	Set 1	Set 2	Set 3	Set 4	Set 5
*P*	([0.25, 0.35], [0.25, 0.35])	([1, 1], [0, 0])	([0.5, 0.5], [0.5, 0.5])	([0.1, 0.1], [0.2, 0.2])	([1, 1], [0, 0])
*Q*	([0.35, 0.45], [0.35, 0.45])	([0, 0], [0, 0])	([0, 0], [0, 0])	([0.3, 0.3], [0.4, 0.4])	([0, 0], [1, 1])
Eq. (9)	0.1	**0.5**	**0.5**	0.2	1.0
Eq. (10)	0.1	0.707	0.5	0.2	1.0
Eq. (11)	0.1	**1.0**	0.5	0.2	**1.0**
Eq. (12)	0.01	**1.0**	0.25	0.04	**1.0**
Eq. (13)	0.173	**1.0**	0.866	0.346	**1.0**
Eq. (14)	0.1	**0.5**	**0.5**	0.2	**0.5**
Eq. (15)	0.1	**0.577**	0.5	0.2	**0.577**
Eq. (16)	0.1	**0.5**	**0.5**	0.2	**0.5**
Eq. (17)	0.05	**0.25**	**0.25**	0.1	**0.25**
Eq. (18)	0.075	**0.375**	**0.375**	0.15	**0.375**
Eq. (19)	0.123	**0.699**	0.616	0.246	**0.699**
Eq. (20)	0.1	**0.5**	**0.5**	0.2	1.0
Proposed-1	0.2	0.5	**1.0**	0.4	**1.0**
Proposed-2	0.19	0.5	0.75	0.36	1.0

Thus, it is worth mentioned that the introduced interval-valued intuitionistic fuzzy distance measure $d_{2} \left( {P,\,Q} \right)$ provides reasonable results for considered IVIFSs, whilst most of the existing interval-valued intuitionistic fuzzy distance measures generate some counter-intuitive cases.

## A hybrid WISP method for interval-valued intuitionistic fuzzy MCDM problems

This section firstly proposes an extended WISP method is introduced for solving the decision-making problems under interval-valued intuitionistic fuzzy environment. Consider $F = \left\{ {F_{1} ,\,F_{2} ,...,\,F_{m} } \right\}$ be a set of options and let $H = \left\{ {H_{1} ,\,H_{2} ,...,\,H_{n} } \right\}$ be a set of criteria. A group of decision experts (DEs) $E = \left\{ {E_{1} ,\,E_{2} ,...,\,E_{t} } \right\}$ is created to choose the most suitable option by means of considered criteria set. The decision experts create a linguistic decision matrix $D\, = \,\left( {\delta_{ij}^{(k)} } \right)_{m \times n}$ in which $\delta_{ij}^{(k)} \, = \,\left\langle {\left[ {\mu_{ij}^{ - (k)} ,\,\mu_{ij}^{ + (k)} } \right],\,\left[ {\nu_{ij}^{ - (k)} ,\,\nu_{ij}^{ + (k)} } \right]} \right\rangle$ denotes the linguistic assessment of alternative *F*_*i*_ by means of *H*_*j*_, where *i* = 1, 2, …, *m*, *j* = 1, 2, …, *n* and *k* = 1, 2,…, *t*. To determine the solution of group decision-making problems, the proposed model includes the following steps:

Step 1: Compute the decision experts’ significance values.
  Consider the linguistic assessments of decision experts’ significance values and convert them into IVIFNs. Let $E_{k} = \,\left( {\left[ {\mu_{k}^{ - } ,\mu_{k}^{ + } } \right],\,\left[ {\nu_{k}^{ - } ,\,\nu_{k}^{ + } } \right]} \right),\,k = 1,2,...,t$ be the performance of *k*^th^ decision expert, where *k* = 1, 2,…, *t*. Then the procedure for estimating the numeric significance value of *k*^th^ decision expert is given by
  21 $$\vartheta_{k} \, = \frac{{\left( {\mu_{k}^{ - } + \,\mu_{k}^{ + } } \right)\left( {2 + \,\pi_{k}^{ - } + \,\pi_{k}^{ + } } \right)}}{{\sum\limits_{k = 1}^{t} {\left( {\left( {\mu_{k}^{ - } + \,\mu_{k}^{ + } } \right)\left( {2 + \,\pi_{k}^{ - } + \,\pi_{k}^{ + } } \right)} \right)} }},\; {\rm where}\;\,\,k = 1, \, 2, \ldots ,t,\,\vartheta_{k} \, \in \left[ {0,\,1} \right]\,\,\;{\text{and}} \;\,\,\sum\limits_{k\, = 1}^{t} {\vartheta_{k} } = \,1.$$
Step 2: Create an aggregated interval-valued intuitionistic fuzzy decision matrix.
  The entire individual experts’ opinions are combined to create an aggregated interval-valued intuitionistic fuzzy decision matrix. For this purpose, an interval-valued intuitionistic fuzzy weighted averaging operator is used to determine the aggregated decision matrix $Z = \left( {z_{ij} } \right)_{m \times \,n} ,$ wherein
  22 $$\begin{gathered} z_{ij} = \left( {\left[ {\mu_{ij}^{ - } ,\,\mu_{ij}^{ + } } \right],\,\left[ {\nu_{ij}^{ - } ,\,\nu_{ij}^{ + } } \right]} \right) = IVIFWA_{{\vartheta_{k} }} \left( {\delta_{ij}^{\left( 1 \right)} ,\,\delta_{ij}^{\left( 2 \right)} ,...,\,\delta_{ij}^{\left( k \right)} } \right)\, \hfill \\ = \left( {\left[ {1 - \prod\limits_{k = 1}^{t} {\left( {1 - \mu_{ij}^{ - \left( k \right)} } \right)^{{\vartheta_{k} }} } ,\,\,1 - \prod\limits_{k = 1}^{t} {\left( {1 - \mu_{ij}^{ + \left( k \right)} } \right)^{{\vartheta_{k} }} } } \right],\left[ {\prod\limits_{k = 1}^{t} {\left( {\nu_{ij}^{ - \left( k \right)} } \right)}^{{\vartheta_{k} }} ,\,\,\prod\limits_{k = 1}^{t} {\left( {\nu_{ij}^{ + \left( k \right)} } \right)}^{{\vartheta_{k} }} } \right]} \right). \hfill \\ \end{gathered}$$
Step 3: Calculate the weights of criteria.
  Let $w = \left\{ {w_{1} ,w_{2} ,...,w_{n} } \right\}$ be criteria weights’ set which satisfies $\sum\limits_{j = 1}^{n} {w_{j} } \, = 1$ and $\,w_{j} \in \left[ {0,\,\,1} \right].$ Next, we present an integrated weighting tool to estimate the criteria weights during the assessment of alternatives.
  **Case I:** Objective weight determination through distance measure-based formula.
    Based on the proposed distance measure, we present a formula to estimate the objective weight of *j*^th^ criterion, given as follows:
    23 $$w_{j}^{o} = \frac{{\frac{1}{m - 1}\sum\limits_{i = 1}^{m} {\sum\limits_{s = 1}^{m} {d_{\alpha} \left( {z_{ij} ,\,z_{sj} } \right)} } }}{{\sum\limits_{j = 1}^{n} {\left( {\frac{1}{m - 1}\sum\limits_{i = 1}^{m} {\sum\limits_{s = 1}^{m} {d_{\alpha} \left( {z_{ij} ,\,z_{sj} } \right)} } } \right)} }},\,\,\,\forall \,j\,\,{\rm and} \,\,\alpha\, = 1,\,2.$$
  **Case II:** Subjective weight through RANCOM model.
    This method considers the following steps for computing the subjective weight of criteria:
Step 4: Based on the experts’ linguistic assessment ratings for the criteria, an aggregated matrix is created using interval-valued intuitionistic fuzzy weighted averaging (IVIFWA) (or interval-valued intuitionistic fuzzy weighted geometric (IVIFWG)) operator.
  24a $$N = \left( {z_{j} } \right)_{1 \times \,n} = IVIFWA_{{\vartheta_{k} }} \left( {z_{j}^{\left( 1 \right)} ,z_{j}^{\left( 2 \right)} ,...,z_{j}^{\left( l \right)} } \right),\,j = 1,2,...,n.$$
  or
  24b $$N = \left( {z_{j} } \right)_{1 \times \,n} = IVIFWG_{{\vartheta_{k} }} \left( {z_{j}^{\left( 1 \right)} ,z_{j}^{\left( 2 \right)} ,...,z_{j}^{\left( l \right)} } \right),\,j = 1,2,...,n.$$
Step 5: Find the score value of each aggregated element based on Eq. (25).
  25 $$\overline{\eta }_{j} = \frac{1}{2}\left( {{\mathbb{S}}\left( {z_{j} } \right)\, + \,1} \right),\,j = 1,2,...,n.$$
Step 6: Define the criteria ranking.
  The decision experts rank the given criteria. The minimum score value represents the most significant rank of the criterion. Some criteria may have equal score values, which means that ties are allowed during the DEs’ judgment.
Step 7: Construct the matrix of ranking comparison.
  Based on the pairwise comparison of the positions of criteria, the matrix of ranking comparison can be represented as
  26 $$\begin{gathered} \begin{array}{*{15}c} {H_{1} } & {\,H_{2} } & {\, \cdots } & {\,\,H_{n} } \\ \end{array} \hfill \\ MRC = \begin{array}{*{20}c} {H_{1} } \\ {H_{2} } \\ \vdots \\ {H_{n} } \\ \end{array} \left[ {\begin{array}{*{20}c} {\varphi_{11} } & {\varphi_{11} } & \cdots & {\varphi_{1n} } \\ {\varphi_{21} } & {\varphi_{22} } & \cdots & {\varphi_{2n} } \\ \vdots & \vdots & \ddots & \vdots \\ {\varphi_{n1} } & {\varphi_{n2} } & \cdots & {\varphi_{nn} } \\ \end{array} } \right],\;\;\;{\text{where}}\;\;\varphi_{tj} = \left\{ \begin{gathered} 1,\,\,\,\,\,\,\,\,if\,\xi \left( {H_{j} } \right) < \xi \left( {H_{l} } \right), \hfill \\ 0.5,\,\,\,if\,\xi \left( {H_{j} } \right) < \xi \left( {H_{l} } \right), \hfill \\ 0,\,\,\,\,\,\,\,if\,\xi \left( {H_{j} } \right) > \xi \left( {H_{l} } \right), \hfill \\ \end{gathered} \right. \hfill \\ \end{gathered}$$
  and $\xi \left( {H_{j} } \right)$ is a significance function of *j*th criterion.
Step 8: Compute the summed criteria weights.
  In accordance with previous step, the horizontal vector of the summed criteria weights is computed using Eq. (27).
  27 $$SCW_{j} = \sum\limits_{t = 1}^{n} {\varphi_{tj} } ,\,j = 1,2,...,n.$$
Step 9: Derive the subjective weight of *j*th criterion.
  Thus, the subjective weight of *j*th criterion is defined as
  28 $$w_{j}^{s} = \frac{{SCW_{j} }}{{\sum\nolimits_{j = 1}^{n} {SCW_{j} } }},\,j = 1,2,...,n.$$
  **Case III**: Integrated interval-valued intuitionistic fuzzy-distance measure-RANCOM weighting tool.
    In order to consider the advantages of objective and subjective weights of criteria through distance measure-based formula and RANCOM model, respectively, we present an integrated weighting formula, given as
    29 $$w_{j} = \zeta w_{j}^{o} + \left( {1 - \,\zeta } \right)w_{j}^{s} ,$$
    where $\zeta \, \in \,\left[ {0,\,1} \right]$ represents the precision factor of decision strategy.
Step 10: Normalize the aggregated interval-valued intuitionistic fuzzy decision matrix.
  The aggregated interval-valued intuitionistic fuzzy decision matrix is normalized through linear and vector normalization tools as
Step 11: It eradicates the dimensions of criteria based on the principle of max–min operator. A linear normalized aggregated interval-valued intuitionistic fuzzy decision matrix ${\mathbb{N}}^{\left( 1 \right)} \, = \,\left( {\varepsilon_{ij}^{\left( 1 \right)} } \right)_{m \times n}$ is created using Eq. (30), where
  30 $$\varepsilon_{ij}^{\left( 1 \right)} = \left( {\left[ {\overline{\mu }_{ij}^{ - \left( 1 \right)} ,\overline{\mu }_{ij}^{ + \left( 1 \right)} } \right],\left[ {\overline{\nu }_{ij}^{ - \left( 1 \right)} ,\overline{\mu }_{ij}^{ + \left( 1 \right)} } \right]} \right) = \frac{{z_{ij} }}{{\max_{i} {\mathbb{S}}\left( {z_{ij} } \right)}}.$$
Step 12: A vector normalized aggregated interval-valued intuitionistic fuzzy decision matrix ${\mathbb{N}}^{\left( 2 \right)} \, = \,\left( {\varepsilon_{ij}^{\left( 2 \right)} } \right)_{m \times n}$ is constructed using Eq. (31), where
  31 $$\varepsilon_{ij}^{\left( 2 \right)} = \left( {\left[ {\overline{\mu }_{ij}^{ - \left( 2 \right)} ,\overline{\mu }_{ij}^{ + \left( 2 \right)} } \right],\left[ {\overline{\nu }_{ij}^{ - \left( 2 \right)} ,\overline{\mu }_{ij}^{ + \left( 2 \right)} } \right]} \right) = \frac{{z_{ij} }}{{\sqrt {\sum\nolimits_{i = 1}^{m} {\left( {{\mathbb{S}}\left( {z_{ij} } \right)} \right)^{2} } } }}.$$
Step 13: Find the averaged normalized aggregated interval-valued intuitionistic fuzzy decision matrix.
  The averaged normalized aggregated interval-valued intuitionistic fuzzy decision matrix ${\mathbb{N}} = \left( {\varepsilon_{ij} } \right)_{m\, \times \,n} ,$ where $\varepsilon_{ij} = \left( {\left[ {\overline{\mu }_{ij}^{ - } ,\overline{\mu }_{ij}^{ + } } \right],\left[ {\overline{\nu }_{ij}^{ - } ,\overline{\mu }_{ij}^{ + } } \right]} \right)$ is computed using Eq. (32).
  32 $$\varepsilon_{ij} = \beta \varepsilon_{ij}^{\left( 1 \right)} + \left( {1 - \beta } \right)\varepsilon_{ij}^{\left( 2 \right)} ,$$
  where $\varepsilon_{ij}$ denotes the averaged normalized aggregated interval-valued intuitionistic fuzzy number. *β* is a normalization parameter changing from 0 to 1. Here, we take *β* = 0.5.
Step 14: Determine the measures through weighted sum deviation and weighted sum ratio by means of linear normalization formula.
  To find the measures, we first compute the collective assessment degree using interval-valued intuitionistic fuzzy weighted averaging operator for benefit and cost criteria as
  33 $$s_{i}^{ + } \, = \left( {\left[ {1 - \prod\limits_{{j \in H_{b} }} {\left( {1 - \overline{\mu }_{ij}^{ - } } \right)^{{w_{j} }} ,1 - \prod\limits_{{j \in H_{b} }} {\left( {1 - \overline{\mu }_{ij}^{ + } } \right)^{{w_{j} }} } } } \right],\,\left[ {\prod\limits_{{j \in H_{b} }} {\left( {\overline{\nu }_{ij}^{ - } } \right)^{{w_{j} }} } ,\prod\limits_{{j \in H_{b} }} {\left( {\overline{\nu }_{ij}^{ + } } \right)^{{w_{j} }} } } \right]} \right),\ \,i=1,2,...,m.$$
  34 $$s_{i}^{ - } \, = \,\left( {\left[ {1 - \prod\limits_{{j \in H_{n} }} {\left( {1 - \overline{\mu }_{ij}^{ - } } \right)^{{w_{j} }} ,1 - \prod\limits_{{j \in H_{n} }} {\left( {1 - \overline{\mu }_{ij}^{ + } } \right)^{{w_{j} }} } } } \right],\,\left[ {\prod\limits_{{j \in H_{n} }} {\left( {\overline{\nu }_{ij}^{ - } } \right)^{{w_{j} }} } ,\prod\limits_{{j \in H_{n} }} {\left( {\overline{\nu }_{ij}^{ + } } \right)^{{w_{j} }} } } \right]} \right), \,i=1,2,...,m..$$
  Based on the score values of collective assessment degree, the measures of weighted sum deviation and weighted sum ratio are computed by Eq. (35).
  35 $$s_{i}^{d} = {\mathbb{S}}\left( {s_{i}^{ + } } \right) - {\mathbb{S}}\left( {s_{i}^{ - } } \right),\,i = 1,2,...,m.$$
  where
  36 $$s_{i}^{r} = \left\{ \begin{gathered} {{{\mathbb{S}}\left( {s_{i}^{ + } } \right)} \mathord{\left/ {\vphantom {{{\mathbb{S}}\left( {s_{i}^{ + } } \right)} {{\mathbb{S}}\left( {s_{i}^{ - } } \right),\,\,if\,\,H_{b} \cap H_{n} \ne \emptyset }}} \right. \kern-0pt} {{\mathbb{S}}\left( {s_{i}^{ - } } \right),\,\,if\,\,H_{b} \cap H_{n} \ne \emptyset }} \hfill \\ {\mathbb{S}}\left( {s_{i}^{ + } } \right),\,\,\,\,if\,\,H_{n} = \emptyset \hfill \\ {1 \mathord{\left/ {\vphantom {1 {{\mathbb{S}}\left( {s_{i}^{ - } } \right)}}} \right. \kern-0pt} {{\mathbb{S}}\left( {s_{i}^{ - } } \right)}},\,\,if\,H_{b} = \emptyset \hfill \\ \end{gathered} \right.$$
Step 15: Determine the measures through weighted product deviation and weighted product ratio by vector normalization tool.
  To find the measures, we first compute the collective assessment degree using interval-valued intuitionistic fuzzy geometric operator for beneficial and non-beneficial criteria as
  37 $$p_{i}^{ + } \, = \left( {\left[ {\prod\limits_{{j \in H_{b} }} {\left( {\overline{\mu }_{ij}^{ - } } \right)^{{w_{j} }} } ,\prod\limits_{{j \in H_{b} }} {\left( {\overline{\mu }_{ij}^{ + } } \right)^{{w_{j} }} } } \right]\left[ {1 - \prod\limits_{{j \in H_{b} }} {\left( {1 - \overline{\nu }_{ij}^{ - } } \right)^{{w_{j} }} ,1 - \prod\limits_{{j \in H_{b} }} {\left( {1 - \overline{\nu }_{ij}^{ + } } \right)^{{w_{j} }} } } } \right]} \right), \, i=1,2,...,m.$$
  38 $$p_{i}^{ - } = \left( {\left[ {\prod\limits_{{j \in H_{n} }} {\left( {\overline{\mu }_{ij}^{ - } } \right)^{{w_{j} }} } ,\prod\limits_{{j \in H_{n} }} {\left( {\overline{\mu }_{ij}^{ + } } \right)^{{w_{j} }} } } \right]\left[ {1 - \prod\limits_{{j \in H_{n} }} {\left( {1 - \overline{\nu }_{ij}^{ - } } \right)^{{w_{j} }} ,1 - \prod\limits_{{j \in H_{n} }} {\left( {1 - \overline{\nu }_{ij}^{ + } } \right)^{{w_{j} }} } } } \right]} \right), i=1,2,...,m.$$
  Based on the score values of collective assessment degree, the measures of weighted product deviation and weighted product ratio are computed by Eq. (39).
  39 $$p_{i}^{d} = {\mathbb{S}}\left( {p_{i}^{ + } } \right) - {\mathbb{S}}\left( {p_{i}^{ - } } \right),$$
  where
  40 $$p_{i}^{r} = \left\{ \begin{gathered} {{{\mathbb{S}}\left( {p_{i}^{ + } } \right)} \mathord{\left/ {\vphantom {{{\mathbb{S}}\left( {p_{i}^{ + } } \right)} {{\mathbb{S}}\left( {p_{i}^{ - } } \right),\,\,if\,\,H_{b} \cap H_{n} \ne \emptyset }}} \right. \kern-0pt} {{\mathbb{S}}\left( {p_{i}^{ - } } \right),\,\,if\,\,H_{b} \cap H_{n} \ne \emptyset }} \hfill \\ {\mathbb{S}}\left( {p_{i}^{ + } } \right),\,\,\,\,if\,\,H_{n} = \emptyset \hfill \\ {1 \mathord{\left/ {\vphantom {1 {{\mathbb{S}}\left( {p_{i}^{ - } } \right)}}} \right. \kern-0pt} {{\mathbb{S}}\left( {p_{i}^{ - } } \right)}},\,if\,\,H_{b} = \emptyset \hfill \\ \end{gathered} \right..$$
Step 16: Calculate the improved utility degree of each alternative by means of Eqs. (41)–(44).
  41 $$u_{i}^{sd} = \frac{{1 + s_{i}^{d} }}{{1 + \max_{i} s_{i}^{d} }},$$
  42 $$u_{i}^{sr} = \frac{{1 + s_{i}^{r} }}{{1 + \max_{i} s_{i}^{r} }},$$
  43 $$u_{i}^{pd} = \frac{{1 + p_{i}^{d} }}{{1 + \max_{i} p_{i}^{d} }},$$
  44 $$u_{i}^{pr} = \frac{{1 + p_{i}^{r} }}{{1 + \max_{i} p_{i}^{r} }}.$$
Step 17: Determine the overall utility degree of each option using Eq. (45).
  45 $$u_{i} = \frac{1}{4}\left( {u_{i}^{sd} + u_{i}^{sr} + u_{i}^{pd} + u_{i}^{pr} } \right),\,\,{\text{where}}\,\,u_{i} \in \left[ {0,1} \right],\,i=1,2,...,m.$$

On the basis of obtained overall utility degrees, rank the alternatives and choose the optimal one.

## Result and discussion

This section firstly presents an empirical study of location selection for OWPS under the context of IVIFSs and further discusses the sensitivity and comparative analyses to demonstrate the robustness of the obtained results.

### Case study: OWPS location selection

In spite of higher costs today, offshore wind energy is important for decarbonizing India’s power sector, delivering a higher capacity utilization factor than onshore farms. India is blessed with a coastline of about 7600 km surrounded by water on three sides and has good prospects of harnessing offshore wind energy. By 2030, the Indian government has set a target to install 30 Gigawatt of offshore wind energy. The country has estimated that the western state Gujarat and southern state Tamil Nadu have approximately 70 Gigawatt of potential for offshore wind energy, which are sufficient to power over 50 million homes. Offshore wind energy is momentous in India’s goal of accomplishing 500 GW of renewable energy resources capacity by 2030 and attaining its target of becoming net zero-carbon measures by 2070. According to Ministry of New and Renewable Energy, competitive tariffs for offshore wind energy can be accomplished through higher productivities of the wind turbines after the development of an ecosystem within the country (https://mnre.gov.in/wind/offshore-wind/^57,58^). Indian government is enthusiastically taking measures to tap into the offshore wind energy potentials of the Indian coast. As a result of the complications of developing offshore wind power station (OWPS) and learnings from other developing markets, it is predicted that local companies will need partnership and capacity building with professional organizations, mainly during the progressive years, to accomplish the target that the government has set.

The state of Gujarat is blessed with a long coastline of 1600 km, where the wind speeds are adequate for conversion into electrical energy. This state has got the highest potentiality for setting up the OWPS location in the country, even though the intensity of wind in the state is at medium and low level in comparison to that of other states in the south India. Consequently, many experts have invited to evaluate the locations for OWPS in Gujarat (India). Here, the considered OWPS location alternatives are site-1, site-2, site-3, site-4 and site-5. For this purpose, a panel of three experts is created to enhance the offshore wind energy generation capacity in the country. Initially, the decision experts provided her/his rating qualitatively using nine points Likert scales. In addition, the criteria that may have an effect on the OWPS location selection are assembled through literature survey. Table 4 presents the description of each considered criterion. Here, *H*_1_, *H*_2_, *H*_3_ and *H*_6_ are cost criteria and others are benefit criteria.

**Table 4:** Considered criteria for OWPS location selection.

Dimension	Criteria	Type	References
Risk aspect	Natural disaster (*H*_1_)	Cost	^40^
	Monitoring risk (*H*_2_)	Cost	^40^
Economic aspect	Construction, operation and maintenance cost (*H*_3_)	Cost	^5,4^
Construction conditions aspect	Construction materials (*H*_4_)	Benefit	^40^
	Grid connection conditions (*H*_5_)	Benefit	^39,4,40^
Environmental aspect	Ecological environment impact (*H*_6_)	Cost	^35^
Wind resources aspect	Wind speed (*H*_7_)	Benefit	^58,40^
	Wind energy density (*H*_8_)	Benefit	^35,39^
	Stable prevailing wind direction (*H*_9_)	Benefit	^5,39,58,40^

The proposed method is applied on the given decision-making problem and the required implementation procedure is presented in the following steps:

*Steps 1–2:* Table 5 presents the linguistic variables and the consequent interval-valued intuitionistic fuzzy numbers^46^. With the use of Table 5 and Eq. (21), the weight value of each decision expert is computed and shown in Table 6.

**Table 5:** Ratings for OWPS location selection.

LVs	IVIFNs
Absolutely significant (AS)	([0.9,0.95], [0,0.05])
Very significant (VS)	([0.8,0.9], [0.05,0.1])
Significant (S)	([0.7,0.8], [0.1,0.15])
Slight significant (SS)	([0.65,0.7], [0.15,0.2])
Average (A)	([0.5,0.6], [0.20,0.35])
Slight insignificant (SI)	([0.4,0.5], [0.4,0.45])
Insignificant (I)	([0.25,0.4], [0.45,0.5])
Very insignificant (VI)	([0.15,0.2], [0.6,0.75])
Extremely insignificant (EI)	([0.05,0.1], [0.8,0.9])

**Table 6:** Decision experts’ significance values for OWPS locations evaluation.

Aspects	*E*_1_	*E*_2_	*E*_3_
Linguistic ratings	S	AS	VS
IVIFNs	([0.7,0.8], [0.1,0.15])	([0.9,0.95], [0,0.05])	([0.8,0.9], [0.05, 0.1])
Score value	3.3750	3.8850	3.6550
Weight	0.3092	0.3559	0.3349
  Considering the linguistic scales into mind, the DEs provide their opinions for each OWPS location alternative with respect to the considered criteria and then the required linguistic decision matrix is constructed in Table 7. To create the aggregated interval-valued intuitionistic fuzzy decision matrix, the interval-valued intuitionistic fuzzy weighted averaging operator Eq. (22) is applied on linguistic decision matrix and the required results are presented in Table 8 for OWPS location alternatives assessment.

**Table 7:** Linguistic decision matrix for assessing the OWPS locations given by decision experts.

Criteria	*F*_1_	*F*_2_	*F*_3_	*F*_4_	*F*_5_
*H*_1_	(I,VI,SI)	(EI,I,VI)	(I,VI,SI)	(VI,EI,SI)	(VI,I,SI)
*H*_2_	(I,VI,I)	(SI,VI,A)	(A,SI,EI)	(I,SI,I)	(I,I,I)
*H*_3_	(VI,I,SI)	(SI,I,I)	(VI,I,I)	(VI,I,VI)	(VI, VI,SI)
*H*_4_	(VS,VS,S)	(VS,A,S)	(A,VS,A)	(A,S,SS)	(VS,SI,SS)
*H*_5_	(A,SS,VS)	(VS,SI,S)	(S,VS,SS)	(SS,VS,S)	(VS,SS,VS)
*H*_6_	(VI,I,SI)	(SI,EI,EI)	(VI,SI,I)	(VI,I,I)	(VI,SI,EI)
*H*_7_	(VS,S,S)	(A,SS,VS)	(SS,VS,SS)	(VS,VS,SS)	(VS,A,SI)
*H*_8_	(A,S,SI)	(SS,VS,S)	(S,S,A)	(S,VS,A)	(S,VS,A)
*H*_9_	(S,VS,S)	(VS,VS,VS)	(A,A,VS)	(A,SS,A)	(VS,VS,S)

**Table 8:** Aggregated matrix for assessing the OWPS locations given by experts.

Criteria	*F*_1_	*F*_2_	*F*_3_	*F*_4_	*F*_5_
*H*_1_	([0.272, 0.375], [0.479, 0.558])	([0.159, 0.251], [0.592, 0.687])	([0.272, 0.375], [0.479, 0.558])	([0.213, 0.287], [0.580, 0.674])	([0.277, 0.383], [0.473, 0.547])
*H*_2_	([0.216, 0.335], [0.499, 0.578])	([0.361, 0.452], [0.366, 0.496])	([0.339, 0.432], [0.407, 0.525])	([0.307, 0.438], [0.432, 0.482])	([0.250, 0.400], [0.450, 0.500])
*H*_3_	([0.277, 0.383], [0.473, 0.547])	([0.300, 0.433], [0.434, 0.484])	([0.220, 0.344], [0.492, 0.567])	([0.187, 0.278], [0.542, 0.649])	([0.244, 0.317], [0.524, 0.632])
*H*_4_	([0.771, 0.874], [0.063, 0.115])	([0.683, 0.793], [0.103, 0.179])	([0.639, 0.756], [0.122, 0.224])	([0.630, 0.716], [0.142, 0.215])	([0.643, 0.744], [0.151, 0.215])
*H*_5_	([0.676, 0.773], [0.113, 0.189])	([0.661, 0.776], [0.132, 0.196])	([0.727, 0.821], [0.090, 0.143])	([0.728, 0.823], [0.089, 0.142])	([0.756, 0.852], [0.074, 0.128])
*H*_6_	([0.277, 0.383], [0.473, 0.547])	([0.176, 0.250], [0.646, 0.726])	([0.280, 0.385], [0.472, 0.546])	([0.220, 0.344], [0.492, 0.567])	([0.221, 0.296], [0.572, 0.665])
*H*_7_	([0.735, 0.839], [0.081, 0.132])	([0.676, 0.773], [0.113, 0.189])	([0.713, 0.797], [0.101, 0.156])	([0.759, 0.856], [0.072, 0.126])	([0.600, 0.719], [0.164, 0.258])
*H*_8_	([0.557, 0.663], [0.197, 0.282])	([0.728, 0.823], [0.089, 0.142])	([0.644, 0.748], [0.126, 0.199])	([0.692, 0.803], [0.099, 0.172])	([0.692, 0.803], [0.099, 0.172])
*H*_9_	([0.740, 0.844], [0.078, 0.130])	([0.800, 0.900], [0.050, 0.100])	([0.632, 0.749], [0.126, 0.230])	([0.560, 0.639], [0.181, 0.287])	([0.771, 0.874], [0.063, 0.115])
*Step 3:* Applying Eq. (23), we determine the discrimination using proposed distance measure of aggregated IVIFNs given in Table 8. Next, we derive the objective weight of criteria for OWPS location selection and shown as
  $$w_{j}^{o} = \left\{ {0.1144, \, 0.0988, \, 0.112, \, 0.0973, \, 0.0655, \, 0.1285, \, 0.105, \, 0.1092, \, 0.1693} \right\}.$$
  Next, to determine the subjective weight of criteria through RANCOM model, we compute the aggregated value of individual experts’ opinions and then determine their score value based on Eq. (24) and Eq. (25), respectively. The required results are presented in Table 9. Based on the comparisons made by experts, the matrix of ranking comparison is determined using Eq. (26) and presented in Table 10. Based on the matrix of ranking comparison, the summed criteria weights are calculated using Eq. (27) and given in last column in Table 10. From Eq. (28), we have calculated the subjective weight of each criterion for OWPS location selection. The required subjective weight set is
  $$w_{j}^{s} = \left\{ {0.0123, \, 0.0370, \, 0.1111, \, 0.0617, \, 0.0864, \, 0.1358, \, 0.1852, \, 0.1605, \, 0.2099} \right\}.$$

**Table 9:** Aggregated opinions and their score value.

Criteria	*E*_1_	*E*_2_	*E*_3_	IVIFNs	Score value	Rank
*H*_1_	SI	S	A	([0.559, 0.665], [0.194, 0.298])	0.683	9
*H*_2_	S	A	A	([0.573, 0.677], [0.161, 0.269])	0.705	8
*H*_3_	A	SS	S	([0.629, 0.714], [0.143, 0.216])	0.746	5
*H*_4_	VS	A	SI	([0.600, 0.719], [0.164, 0.258])	0.724	7
*H*_5_	S	S	SI	([0.622, 0.728], [0.159, 0.217])	0.743	6
*H*_6_	A	S	S	([0.649, 0.752], [0.124, 0.195])	0.771	4
*H*_7_	VS	A	S	([0.683, 0.793], [0.103, 0.179])	0.798	2
*H*_8_	SS	S	SS	([0.669, 0.740], [0.130, 0.181])	0.775	3
*H*_9_	S	VS	VS	([0.773, 0.876], [0.062, 0.113])	0.869	1

**Table 10:** Matrix of ranking comparison and summed criteria weights.

Criteria	Matrix of ranking comparison									*SCW*_*j*_	$w_{j}^{s}$
	*H*_*1*_	*H*_*2*_	*H*_*3*_	*H*_*4*_	*H*_*5*_	*H*_*6*_	*H*_*7*_	*H*_*8*_	*H*_*9*_		
*H*_1_	0.5	0	0	0	0	0	0	0	0	0.5	0.0123
*H*_2_	1	0.5	0	0	0	0	0	0	0	1.5	0.0370
*H*_3_	1	1	0.5	1	1	0	0	0	0	4.5	0.1111
*H*_4_	1	1	0	0.5	0	0	0	0	0	2.5	0.0617
*H*_5_	1	1	0	1	0.5	0	0	0	0	3.5	0.0864
*H*_6_	1	1	1	1	1	0.5	0	0	0	5.5	0.1358
*H*_7_	1	1	1	1	1	1	0.5	1	0	7.5	0.1852
*H*_8_	1	1	1	1	1	1	0	0.5	0	6.5	0.1605
*H*_9_	1	1	1	1	1	1	1	1	0.5	8.5	0.2099
  From Eq. (29), we integrate the distance measure-based tool and the RANCOM model. The integrated weight with the combination of the distance measure and RANCOM model ($\tau =0.5)$ for criteria weights is depicted in the Fig. 1 and is given by
  $$w_{j} = \, \left( {0.0634, \, 0.0679, \, 0.1116, \, 0.0795, \, 0.0759, \, 0.1321, \, 0.1451, \, 0.1349, \, 0.1896} \right).$$

**Figure 1:**
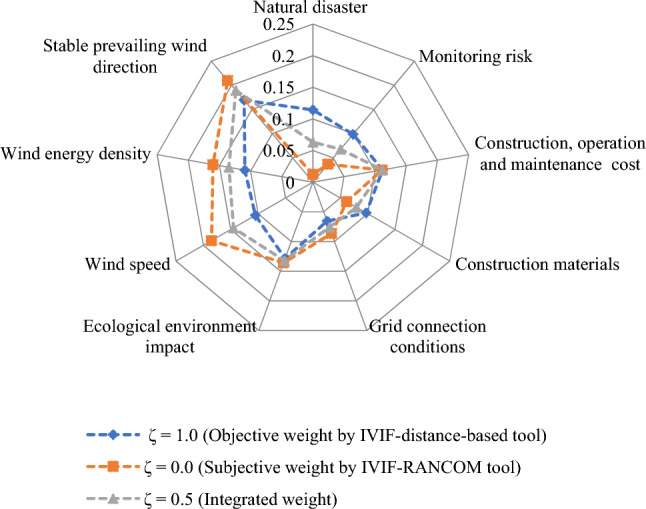
Variation of weights of different criteria for OWPS locations assessment.
  Here, Fig. 1 shows the variation of weight of diverse criteria for OWPS locations evaluation. Stable prevailing wind direction (0.1896) is the most important criterion for OWPS location selection. Wind speed (0.1451) is the second most criteria for OWPS location selection. Wind energy density (0.1349) is third, Ecological environment impact (0.1321) is fourth, Construction, operation and maintenance cost (0.1116) is fifth most important criterion for OWPS locations evaluation and others are considered crucial criterion for OWPS location selection.
*Step 4:* From Table 8 and Eq. (30), Eq. (31), linear normalized and vector normalized aggregated interval-valued intuitionistic fuzzy decision matrices for OWPS locations evaluation are constructed and presented in Tables 11, 12. With the use of Eq. (32), the averaged normalized aggregated interval-valued intuitionistic fuzzy decision matrix for OWPS locations evaluation is determined and presented in Table 13.

**Table 11:** Linear normalization matrix for OWPS location selection.

Criteria	*F*_1_	*F*_2_	*F*_3_	*F*_4_	*F*_5_
*H*_1_	([0.204,0.286], [0.590, 0.658])	([0.116,0.187], [0.687, 0.764])	([0.204,0.286], [0.590, 0.658])	([0.158,0.216], [0.677, 0.754])	([0.207,0.293], [0.584, 0.649])
*H*_2_	([0.142,0.227], [0.644, 0.707])	([0.246,0.316], [0.531, 0.642])	([0.230,0.300], [0.567, 0.666])	([0.207,0.305], [0.588, 0.631])	([0.166,0.276], [0.604, 0.646])
*H*_3_	([0.198,0.280], [0.600, 0.663])	([0.216,0.321], [0.566, 0.610])	([0.156,0.250], [0.617, 0.679])	([0.132,0.199], [0.658, 0.745])	([0.173,0.228], [0.644, 0.732])
*H*_4_	([0.721,0.834], [0.091, 0.153])	([0.630,0.745], [0.140, 0.225])	([0.587,0.705], [0.162, 0.274])	([0.578,0.664], [0.184, 0.263])	([0.591,0.693], [0.195, 0.264])
*H*_5_	([0.617,0.717], [0.157, 0.242])	([0.602,0.721], [0.179, 0.249])	([0.669,0.769], [0.128, 0.191])	([0.670,0.771], [0.127, 0.190])	([0.699,0.804], [0.109, 0.174])
*H*_6_	([0.212,0.299], [0.576, 0.641])	([0.133,0.191], [0.725, 0.790])	([0.215,0.301], [0.575, 0.640])	([0.168,0.267], [0.593, 0.658])	([0.168,0.228], [0.663, 0.740])
*H*_7_	([0.679,0.789], [0.117, 0.178])	([0.618,0.718], [0.156, 0.241])	([0.656,0.744], [0.142, 0.205])	([0.703,0.808], [0.106, 0.171])	([0.542,0.662], [0.214, 0.315])
*H*_8_	([0.491,0.595], [0.260, 0.349])	([0.660,0.762], [0.134, 0.198])	([0.576,0.681], [0.179, 0.262])	([0.624,0.740], [0.146, 0.233])	([0.624,0.740], [0.146, 0.233])
*H*_9_	([0.698,0.807], [0.104, 0.163])	([0.760,0.870], [0.070, 0.130])	([0.588,0.706], [0.159, 0.271])	([0.517,0.595], [0.219, 0.330])	([0.730,0.841], [0.086, 0.146])

**Table 12:** Vector normalization matrix for OWPS location selection.

Criteria	*F*_1_	*F*_2_	*F*_3_	*F*_4_	*F*_5_
*H*_1_	([0.367, 0.492], [0.346, 0.431])	([0.220, 0.341], [0.470, 0.582])	([0.367, 0.492], [0.346, 0.431])	([0.292, 0.386], [0.456, 0.567])	([0.373, 0.501], [0.340, 0.419])
*H*_2_	([0.263, 0.401], [0.417, 0.502])	([0.430, 0.530], [0.283, 0.415])	([0.405, 0.508], [0.324, 0.445])	([0.369, 0.515], [0.348, 0.399])	([0.303, 0.474], [0.367, 0.419])
*H*_3_	([0.362, 0.488], [0.354, 0.434])	([0.390, 0.544], [0.314, 0.366])	([0.292, 0.443], [0.374, 0.455])	([0.249, 0.363], [0.427, 0.550])	([0.321, 0.410], [0.408, 0.529])
*H*_4_	([0.925, 0.974], [0.008, 0.022])	([0.867, 0.938], [0.018, 0.048])	([0.834, 0.916], [0.025, 0.072])	([0.826, 0.891], [0.032, 0.067])	([0.837, 0.909], [0.036, 0.067])
*H*_5_	([0.872, 0.933], [0.019, 0.048])	([0.861, 0.935], [0.025, 0.051])	([0.906, 0.957], [0.012, 0.029])	([0.907, 0.957], [0.012, 0.028])	([0.924, 0.969], [0.009, 0.024])
*H*_6_	([0.374, 0.502], [0.339, 0.418])	([0.244, 0.340], [0.531, 0.630])	([0.378, 0.505], [0.338, 0.417])	([0.302, 0.456], [0.359, 0.440])	([0.302, 0.398], [0.446, 0.554])
*H*_7_	([0.909, 0.962], [0.011, 0.026])	([0.868, 0.931], [0.020, 0.050])	([0.894, 0.943], [0.016, 0.035])	([0.923, 0.969], [0.009, 0.024])	([0.807, 0.898], [0.039, 0.088])
*H*_8_	([0.758, 0.850], [0.059, 0.110])	([0.897, 0.951], [0.015, 0.033])	([0.835, 0.910], [0.027, 0.060])	([0.872, 0.941], [0.018, 0.047])	([0.872, 0.941], [0.018, 0.047])
*H*_9_	([0.913, 0.965], [0.010, 0.025])	([0.946, 0.985], [0.004, 0.015])	([0.837, 0.918], [0.023, 0.070])	([0.774, 0.842], [0.045, 0.104])	([0.931, 0.977], [0.007, 0.020])

**Table 13:** Averaged normalization matrix for OWPS location selection.

Criteria		*F*_2_	*F*_3_	*F*_4_	*F*_5_
*H*_1_	([0.290, 0.398], [0.452, 0.532])	([0.170, 0.268], [0.568, 0.667])	([0.290, 0.398], [0.452, 0.532])	([0.228, 0.306], [0.556, 0.654])	([0.295, 0.406], [0.446, 0.522])
*H*_2_	([0.205, 0.320], [0.518, 0.596])	([0.345, 0.433], [0.388, 0.516])	([0.323, 0.413], [0.428, 0.545])	([0.293, 0.419], [0.452, 0.502])	([0.238, 0.382], [0.471, 0.520])
*H*_3_	([0.284, 0.393], [0.461, 0.536])	([0.308, 0.444], [0.422, 0.472])	([0.227, 0.353], [0.480, 0.556])	([0.193, 0.286], [0.531, 0.640])	([0.251, 0.325], [0.513, 0.622])
*H*_4_	([0.856, 0.934], [0.027, 0.058])	([0.778, 0.874], [0.051, 0.104])	([0.738, 0.843], [0.063, 0.140])	([0.729, 0.809], [0.077, 0.132])	([0.742, 0.833], [0.084, 0.133])
*H*_5_	([0.778, 0.862], [0.054, 0.107])	([0.765, 0.865], [0.067, 0.113])	([0.823, 0.900], [0.040, 0.074])	([0.824, 0.901], [0.039, 0.073])	([0.848, 0.922], [0.031, 0.064])
*H*_6_	([0.297, 0.410], [0.442, 0.518])	([0.190,0.269], [0.621, 0.706])	([0.301, 0.412], [0.441, 0.517])	([0.238,0.369], [0.461, 0.538])	([0.238, 0.318], [0.544, 0.640])
*H*_7_	([0.829, 0.911], [0.035, 0.068])	([0.776, 0.860], [0.056, 0.109])	([0.809, 0.880], [0.048, 0.085])	([0.848, 0.923], [0.031, 0.064])	([0.703, 0.815], [0.091, 0.166])
*H*_8_	([0.649, 0.754], [0.124, 0.196])	([0.813, 0.892], [0.044, 0.081])	([0.735, 0.830], [0.070, 0.125])	([0.780, 0.876], [0.051, 0.104])	([0.780, 0.876], [0.051, 0.104])
*H*_9_	([0.838, 0.918], [0.032, 0.063])	([0.886, 0.955], [0.017, 0.045])	([0.741, 0.845], [0.061, 0.137])	([0.670, 0.747], [0.099, 0.185])	([0.863, 0.939], [0.024, 0.054])
*Step 5:* Table 14 presents the weighted interval-valued intuitionistic fuzzy value of each location using Eq. (33), Eq. (34) by means of interval-valued intuitionistic fuzzy weighted averaging operator. The measures of weighted sum deviation and weighted sum ratio, and their corresponding ranks are computed through Eq. (35), Eq. (36).

**Table 14:** Computed measures of weighted sum deviation and weighted sum ratio for OWPS location selection.

Alternatives	$s_{i}^{ + }$	$s_{i}^{ - }$	${\mathbb{S}}\left( {s_{i}^{ + } } \right)$	${\mathbb{S}}\left( {s_{i}^{ - } } \right)$	$s_{i}^{d}$	Rank	$s_{i}^{r}$	Rank
*F*_1_	([0.638, 0.750], [0.144, 0.216])	([0.638, 0.750], [0.144,0.216])	0.757	0.182	0.575	3	4.160	3
*F*_2_	([0.664, 0.777], [0.127, 0.201])	([0.664, 0.777], [0.127,0.201])	0.778	0.166	0.613	1	4.699	2
*F*_3_	([0.600, 0.707], [0.165, 0.254])	([0.600, 0.707], [0.165,0.254])	0.722	0.186	0.536	4	3.889	5
*F*_4_	([0.584, 0.680], [0.180, 0.263])	([0.584, 0.680], [0.180,0.263])	0.705	0.170	0.535	5	4.139	4
*F*_5_	([0.638, 0.754], [0.145, 0.223])	([0.638, 0.754], [0.145,0.223])	0.756	0.161	0.596	2	4.706	1
*Step 6:* On the similar line, Table 15 displays the weighted interval-valued intuitionistic fuzzy value of each location using Eq. (37), Eq. (38) by means of interval-valued intuitionistic fuzzy weighted geometric operator. The measures of weighted product deviation and weighted product ratio, and their corresponding ranks are determined through Eq. (39), Eq. (40).

**Table 15:** Computed measures of weighted product deviation and weighted product ratio for OWPS location selection.

Alternatives	$p_{i}^{ + }$	$p_{i}^{ - }$	${\mathbb{S}}\left( {p_{i}^{ + } } \right)$	${\mathbb{S}}\left( {p_{i}^{ - } } \right)$	$p_{i}^{d}$	Rank	$p_{i}^{r}$	Rank
*F*_1_	([0.858, 0.917], [0.036,0.065])	([0.618, 0.702], [0.207,0.251])	0.919	0.715	0.203	3	1.284	4
*F*_2_	([0.878, 0.934], [0.027,0.054])	([0.592, 0.673], [0.237,0.292])	0.933	0.684	0.249	1	1.364	1
*F*_3_	([0.843, 0.906], [0.037,0.075])	([0.623, 0.706], [0.200,0.248])	0.909	0.720	0.189	5	1.262	5
*F*_4_	([0.828, 0.884], [0.045,0.083])	([0.588, 0.681], [0.215,0.265])	0.896	0.697	0.199	4	1.285	3
*F*_5_	([0.861, 0.922], [0.035,0.066])	([0.598, 0.673], [0.231,0.287])	0.920	0.688	0.232	2	1.338	2
*Step 7:* Using Eq. (41)–Eq. (44), we estimate the improved utility degree for each OWPS location alternative and the required result is presented in Table 16.

**Table 16:** Different overall utility degrees and ranking for OWPS locations.

Options	$u_{i}^{sd}$	$u_{i}^{sr}$	$u_{i}^{pd}$	$u_{i}^{pr}$	$u_{i}$	Ranking
*F*_1_	0.977	0.904	0.964	0.966	0.9527	3
*F*_2_	1.000	0.999	1.000	1.000	0.9997	1
*F*_3_	0.953	0.857	0.952	0.957	0.9296	5
*F*_4_	0.952	0.901	0.960	0.967	0.9447	4
*F*_5_	0.989	1.000	0.987	0.989	0.9913	2
*Step 8–9:* The measures of overall utility degrees for OWPS location alternatives are calculated by Eq. (45). From Table 16, the option “site-2 (*F*_2_)” is the most appropriate alternative among the other OWPS locations.

### Sensitivity analysis

**Figure 2:**
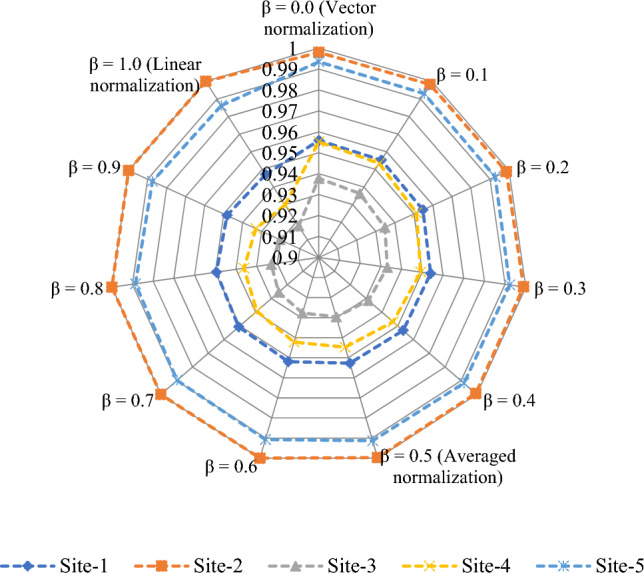
Sensitivity test on normalization parameter for OWPS locations evaluation.
**Case-II:** In this case, we analyze the effect of changing values of decision strategy parameter ‘ζ’. The varying values of ζ help us to assess the sensitivity of the proposed approach with respect to the decision strategy parameter. Table 18 and Fig. 3 represent the sensitivity results for OWPS locations evaluation by means of different values of decision strategy parameter ζ. Using the objective weighting model through distance measure (i.e., ζ = 1.0 in Eq. (29)), the overall utility degree and ranking order of OWPS location alternatives are presented as follows: *F*_1_ = 0.9572, *F*_2_ = 1*.*00, *F*_3_ = 0.9251, *F*_4_ = 0.9346, *F*_5_ = 0.9845 and *F*_2_ $\succ$* F*_5_ $\succ$* F*_1_ $\succ$* F*_4_ $\succ$* F*_3_. Using the subjective weight through RANCOM model (i.e. ζ = 0.0 in Eq. (29)), the overall utility degree and ranking order of OWPS location alternatives are presented as follows: *F*_1_ = 0.9402, *F*_2_ = 0.9918, *F*_3_ = 0.9272, *F*_4_ = 0.9493, *F*_5_ = 0.9938 and *F*_5_ $\succ$* F*_2_ $\succ$* F*_4_ $\succ$* F*_1_ $\succ$* F*_3_. Moreover, using Eq. (29) for ζ = 0.5, we find the same ranking order of OWPS locations as the ranking order obtained by putting ζ = 1.0 in Eq. (29), which is *F*_2_ $\succ$* F*_5_ $\succ$* F*_1_ $\succ$* F*_4_ $\succ$* F*_3_ and thus, the alternative “site-2 (*F*_2_)” is the most suitable choice among all the alternatives for OWPS location selection. Therefore, it is observed that the results obtained by the proposed approach are stable by means of varied values of decision strategy parameter ζ. Thus, it is determined that the changes in decision strategy parameter will enhance the performance of introduced weighting method.

In the current part of the study, we perform the sensitivity analysis with respect to different values of normalization and decision strategy parameters. For this purpose, we present two cases:

**Case-I:** Here, we analyze the effect of changing values of parameter ‘$\beta$’. The varying values of $\beta$ help us to assess the sensitivity of the proposed model with respect to the normalization types. Table 17 and Fig. 2 represent the sensitivity results for OWPS locations evaluation by means of different values of normalization parameter $\beta$. When $\beta$ = 0.0 to $\beta$ =1.0, we find the same preference order of OWPS location alternatives, which is *F*_2_ $\succ$* F*_5_ $\succ$* F*_1_ $\succ$* F*_4_ $\succ$* F*_3_ and thus, the alternative “site-2 (*F*_2_)” is the most suitable choice among all the location alternatives. Thus, it is observed that the results obtained by the developed approach are stable by means of varied values of normalization parameter $\beta$.

**Table 17:** The overall utility degrees of options over normalization parameter.

	$\beta$= 0.0	$\beta$= 0.1	$\beta$= 0.2	$\beta$= 0.3	$\beta$= 0.4	$\beta$= 0.5	$\beta$= 0.6	$\beta$= 0.7	$\beta$= 0.8	$\beta$= 0.9	$\beta$= 1.0
*F*_1_	0.9559	0.9553	0.9547	0.9540	0.9533	0.9527	0.9519	0.9507	0.9495	0.9483	0.9471
*F*_2_	0.9980	0.9983	0.9986	0.9989	0.9993	0.9997	1.0000	1.0000	1.0000	1.0000	1.0000
*F*_3_	0.9378	0.9363	0.9347	0.9331	0.9314	0.9296	0.9277	0.9254	0.9230	0.9205	0.9179
*F*_4_	0.9553	0.9534	0.9513	0.9492	0.9470	0.9447	0.9422	0.9392	0.9362	0.9331	0.9298
*F*_5_	0.9935	0.9931	0.9927	0.9922	0.9918	0.9913	0.9907	0.9897	0.9886	0.9875	0.9863

**Table 18:** The overall utility degrees of options over weighting parameter (ζ) for OWPS location selection.

	ζ = 0.0	ζ = 0.1	ζ = 0.2	ζ = 0.3	ζ = 0.4	ζ = 0.5	ζ = 0.6	ζ = 0.7	ζ = 0.8	ζ = 0.9	ζ = 1.0
*F*_1_	0.9402	0.9431	0.9458	0.9483	0.9506	0.9527	0.9539	0.9547	0.9556	0.9564	0.9572
*F*_2_	0.9918	0.9937	0.9955	0.9970	0.9984	0.9997	1.0000	1.0000	1.0000	1.0000	1.0000
*F*_3_	0.9272	0.9280	0.9287	0.9291	0.9294	0.9296	0.9290	0.9280	0.9271	0.9261	0.9251
*F*_4_	0.9493	0.9485	0.9477	0.9467	0.9457	0.9447	0.9428	0.9407	0.9387	0.9366	0.9346
*F*_5_	0.9938	0.9933	0.9928	0.9923	0.9918	0.9913	0.9900	0.9885	0.9871	0.9858	0.9845

**Figure 3:**
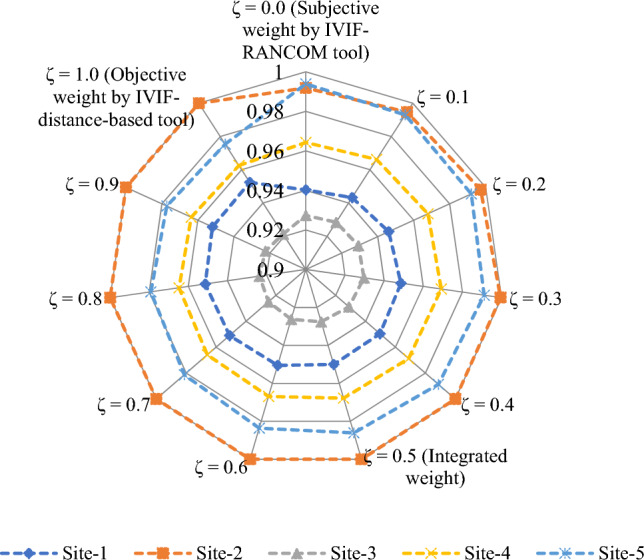
Sensitivity test on weighting parameter for OWPS locations evaluation.

### Comparative analysis

In this subsection, we compare the results of the proposed method and some of the extant methods given by Mishra and Rani^59^, Wang et al.^60^ and Nguyen^61^.

#### Mishra and Rani’s method^59^

The weighted aggregated sum product assessment (WASPAS) method given by Mishra and Rani^59^ is executed on aforesaid OWPS location selection problem. Using this model, the measures obtained through weighted sum model are ([0.609, 0.722], [0.160, 0.236]), $$C_{2}^{(1)} \, =$$([0.661, 0.771], [0.128, 0.201]), $$C_{3}^{(1)} \, =$$([0.581, 0.687], [0.175, 0.265]), $$C_{4}^{(1)} \, =$$([0.590, 0.693], [0.168, 0.256]), $$C_{5}^{(1)} \, =$$([0.618, 0.733], [0.157, 0.236]). In addition, their score values are computed as $${\mathbb{S}}\left( {C_{1}^{(1)} } \right)$$ = 0.734, $${\mathbb{S}}\left( {C_{2}^{(1)} } \right) =$$ 0.775, $${\mathbb{S}}\left( {C_{3}^{(1)} } \right) =$$ 0.707, $${\mathbb{S}}\left( {C_{4}^{(1)} } \right) =$$ 0.715 and $${\mathbb{S}}\left( {C_{5}^{(1)} } \right)\, =$$ 0.740. The measures obtained through weighted product model are $$C_{1}^{(2)} \, =$$ ([0.536, 0.643], [0.223, 0.301]),$$C_{2}^{(2)} \, =$$ ([0.608, 0.716], [0.173, 0.244]), $$C_{3}^{(2)} \, =$$([0.526, 0.633], [0.222, 0.312]), $$C_{4}^{(2)} \, =$$ ([0.519, 0.620], [0.225, 0.315]), $$C_{5}^{(2)} \, =$$([0.547, 0.656], [0.220, 0.300]). Further, their score values are determined as $${\mathbb{S}}\left( {C_{1}^{(2)} } \right) =$$ 0.664, $${\mathbb{S}}\left( {C_{2}^{(2)} } \right) =$$ 0.726, $${\mathbb{S}}\left( {C_{3}^{(2)} } \right) =$$ 0.656, $${\mathbb{S}}\left( {C_{4}^{(2)} } \right) =$$ 0.650 and $${\mathbb{S}}\left( {C_{5}^{(2)} } \right)\, =$$ 0.671. Next, the utility degree of each OWPS location option is computed as $$C_{1} =$$ 0.6988, $$C_{2} =$$ 0.751, $$C_{3} =$$ 0.6817, $$C_{4} =$$ 0.6821 and $$C_{5} =$$ 0.7052. Then the ranking order of OWPS locations is $$F_{2} \succ \,F_{5} \succ \,F_{1} \succ \,F_{4} \succ 
\,F_{3}$$ and the most appropriate alternative is “site-2 (*F*_2_)”.

#### Wang et al.’s method^60^

The complex proportional assessment (COPRAS)^60^ is applied on the OWPS locations evaluation problem, given in "Case study: OWPS location selection" section. The sum of maximization criteria is obtained as $\wp_{1} =$([0.636, 0.749], [0.145, 0.217]), $\wp_{2} =$ ([0.662, 0.775], [0.129, 0.203]), $\wp_{3} =$ ([0.599, 0.705], [0.166, 0.255]), $\wp_{4} =$([0.603, 0.706], [0.166, 0.251]), $\wp_{5} =$ ([0.635, 0.752], [0.147, 0.225]) and the sum of minimization criteria is estimated as $\Im_{1} =$([0.636, 0.749], [0.145,0.217]), $\Im_{2} =$ ([0.662, 0.775], [0.129,0.167]), $\Im_{3} =$([0.599, 0.705], [0.166,0.255]), $\Im_{4} =$ ([0.603, 0.706], [0.166,0.251]), $\Im_{5} =$([0.635, 0.752], [0.147, 0.225]). The relative degrees of OWPS location alternatives are $\ell_{1} =$ 0.4592, $\ell_{2} =$ 0.4786, $\ell_{3} =$ 0.4403, $\ell_{4} =$ 0.4528 and $\ell_{5} =$ 0.4685. Finally, the degree of utility of each option is obtained as $\eta_{1} =$ 95.95, $\eta_{2} =$ 100.00, $\eta_{3} =$ 92.00, $\eta_{4} =$ 94.62 and $\eta_{5} =$ 97.89. Thus, the preference ordering of the OWPS locations is $F_{2} \succ \,F_{5} \succ \,F_{1} \succ \,F_{4} \succ \,F_{3}$ and the “site-2 (*F*_2_)” is considered to be the best choice among the other location candidates.

#### Nguyen’s method^61^

The combined compromise solution (CoCoSo) model is applied on the OWPS locations evaluation problem, given in "Case study: OWPS location selection" section. In this model, the balanced compromise degrees of OWPS location candidates are determined as $Q_{1}^{\left( 1 \right)} =$ 0.1986, $Q_{2}^{\left( 1 \right)} =$ 0.2134, $Q_{3}^{\left( 1 \right)} =$ 0.1937, $Q_{4}^{\left( 1 \right)} =$ 0.1939, $Q_{5}^{\left( 1 \right)} =$ 0.2004, $Q_{1}^{\left( 2 \right)} =$ 2.0591, $Q_{2}^{\left( 2 \right)} =$ 2.2146, $Q_{3}^{\left( 2 \right)} =$ 2.0099, $Q_{4}^{\left( 2 \right)} =$ 2.0103, $Q_{5}^{\left( 2 \right)} =$ 2.0783, $Q_{1}^{\left( 3 \right)} =$ 0.9305, $Q_{2}^{\left( 3 \right)} =$ 1.0, $Q_{3}^{\left( 3 \right)} =$ 0.9077, $Q_{4}^{\left( 3 \right)} =$ 0.9083 and $Q_{5}^{\left( 3 \right)} =$ 0.9391. Next, the overall compromise degrees of OWPS candidates are estimated as *Q*_1_ = 1.7873, *Q*_2_ = 1.9216, *Q*_3_ = 1.7442, *Q*_4_ = 1.7449 and *Q*_5_ = 1.8039. Then the ranking order of OWPS locations is $F_{2} \succ \,F_{5} \succ \,F_{1} \succ \,F_{4} \succ \,F_{3}$ and thus, the option “site-2 (*F*_2_)” is the most suitable alternative among the others.

**Figure 4:**
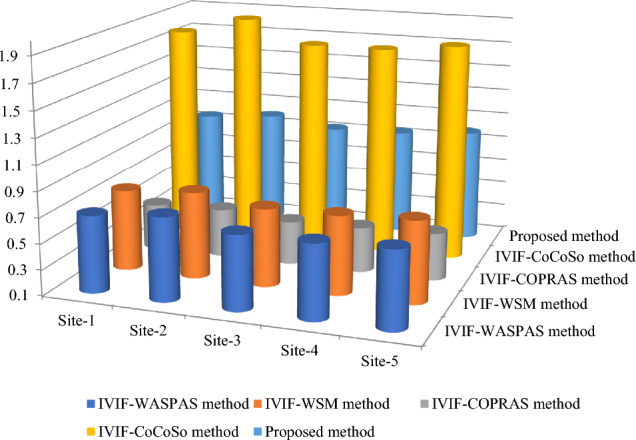
Comparison of introduced model with extant methods for OWPS location selection.

Figure 4 presents the ranking orders of five OWPS locations by introduced and extant MCDM models. From Fig. 3, it can easily be noticed that the most suitable alternative “site-2 (*F*_2_)” is same for all the MCDM approaches including the proposed and extant methods. The main advantages of the proposed MCDM methodology are as follows:

- The distance measure proposed in this study avoids the limitations of existing interval-valued intuitionistic fuzzy distance measures^31–34^ in order to compute the degree of difference between IVIFSs.
- The proposed approach uses the combined weighting procedure based on the distance measure-based model for objective weight of criteria and the RANCOM model for subjective weight of criteria under interval-valued intuitionistic fuzzy environment. While existing MCDM methods by Wang et al.^60^ and Nguyen^61^ considers the direct weights of criteria. Thus, the proposed method provides more accurate results during the assessment of OWPS locations.
- The original WISP approach^27^ uses the single normalization procedure, whereas the proposed WISP method uses the double normalization tools, which evades the difficulty of transforming different dimensions of criteria and the information loss during the locations’ assessment.

### Implication and discussion

**Figure 5:**
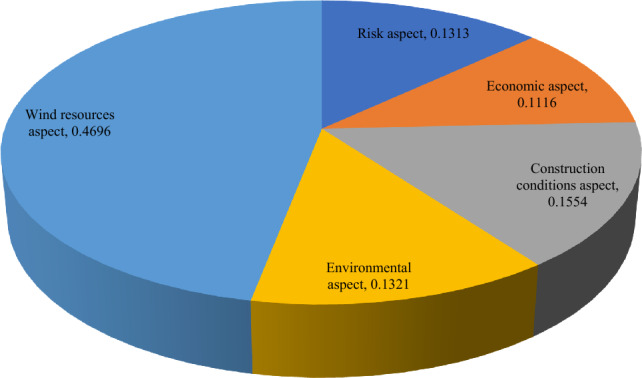
Weights of different dimensions of criteria for OWPS location selection.

During the location selection process of OWPS, wind resources (0.4696) has an important impact on the application and construction of offshore wind power generation projects followed by construction conditions aspect, environmental aspect, risk aspect and economic aspect and presented in Fig. 5. The assessment MCDM model for OWPS location selection, which includes 9 assessment criteria in terms of wind resources, construction conditions, economy, environment and risks aspects. The expert group uses IVIFNs to determine the relative degree of 9 criteria. Based on the experts discussion, it is found that the stable prevailing wind direction (0.1896) is the best criteria and the natural disaster (0.0634) is the worst criteria, shown in Fig. 5. The overall utility degrees of alternatives *F*_1_, *F*_2_, *F*_3_, *F*_4_ and *F*_5_ are 0.9527, 0.9997, 0.9296, 0.9447 and 0.9913, and the prioritization order is *F*_2_ $\succ$* F*_5_ $\succ$* F*_1_ $\succ$* F*_4_ $\succ$* F*_3_. Based on aforementioned discussion, it can be observed that stable prevailing wind direction, wind speed, wind energy density and ecological environment impact are the most impelling factors in evaluating the OWPS location. By means of the concept of introduced framework, we have combined the weight-determining models based on distance measure and the RANCOM, which reduces information loss during the procedure of making decision.

On the basis of above discussed evaluations, five OWPS location options always preserve the prioritization in spite of how criteria weight differs. It can be observed the stability and applicability of the developed model. Though, the proposed model is generally appropriate to the situation where the exact data is fairly ambiguous. Considering realistic situations of several domains, it can be appropriately converted the combination with diverse frameworks.

## Conclusions

In the present work, we have developed a hybrid MCDM framework combining the distance measure, the RANCOM model and the WISP ranking approach for assessing the OWPS locations under interval-valued intuitionistic fuzzy environment. For this purpose, a new distance measure has been proposed to quantify the difference between IVIFSs. Numerical examples have been presented to show the effectiveness of the proposed measures over the existing interval-valued intuitionistic fuzzy distance measures. Further, a hybrid WISP approach has been introduced based on the combination of distance measure, RANCOM model, two normalization tools and interval-valued intuitionistic fuzzy information. In this approach, the criteria weights have been determined based on an integrated formula combining the objective weight through distance measure and the subjective weight through RANCOM model under IVIFSs context. To exemplify the effectiveness of the proposed WISP method, it has been implemented on a case study of OWPS location selection problem from multiple criteria and interval-valued intuitionistic fuzzy information perspectives. In this study, five OWPS locations of Gujarat (India) have extensively been assessed by means of 5 criteria and 9 sub-criteria. Moreover, sensitivity analysis has been discussed with respect to various values of normalization parameter. Furthermore, we have compared the obtained outcomes with some of the existing methods and found that the results are similar and compatible. The advantages of the proposed work include the development of new distance measure, linear and vector normalization tools and criteria weights through integrated weighting model. The findings emphasized the dependability of introduced approach as well as its potential to improve MCDM processes under IVIFS context. Thus, the developed model is more competent and accurate while making decisions in uncertain contexts.

This study has some limitations such as it does not consider the interrelationships among the criteria. In addition, more sustainability indicators should be considered during the assessment of OWPS locations. In future, we will try to overcome the limitations of our present work by developing new model based on Shapley function and evidential reasoning approach under IVIFS environment. Moreover, we will develop new aggregation operators to aggregate the individuals’ information during the assessment of OWPS locations under different uncertain contexts. The possible future research directions can be extended on neutrosophic sets, Plithogenic sets, circular spherical fuzzy sets, linear diophantine sets and hyperbolic sets to rank the OWPS locations.

## Data Availability

All data generated or analyzed during this study are included in this published article.
